# JNJ-79032421, a Novel Membrane-Restricted Mesothelin-Targeting T Cell–Engaging Bispecific Antibody for the Treatment of Mesothelin-Positive Cancers

**DOI:** 10.1158/1535-7163.MCT-25-0665

**Published:** 2026-03-03

**Authors:** Karine Smans, Thomas Nesspor, Sabine De Breucker, Erika van Heerde, Annemie Valckx, Nele Van Slycken, Katrin Sproesser, Bethany Mattson Cypert, Kathryn Packman, Gerald Chu, Jamie Fisher, Nicholas Mazzanti, Ted Petley, Winnie Chan, Brian Del Rosario, Tun Liu, Stuart Ember, Paul L. Shaffer, Jacalyn S. Clawson, Joshua M. Bauml, Sheri Moores, Sylvie Laquerre, Paul R. Gavine

**Affiliations:** 1 Oncology, Johnson & Johnson, Beerse, Belgium.; 2 Therapeutics Discovery, Johnson & Johnson, Springhouse, Pennsylvania.; 3 Oncology, Johnson & Johnson, Springhouse, Pennsylvania.; 4 Oncology, Johnson & Johnson, Cambridge, Massachusetts.

## Abstract

Mesothelin (MSLN) is a cell membrane–anchored glycoprotein overexpressed in several cancers, including pancreatic cancer, ovarian cancer, and mesothelioma. MSLN is protease-cleaved at the membrane proximal region, releasing shed MSLN (sMSLN) into the tumor microenvironment, leaving behind a membrane-bound stub. sMSLN in the tumor microenvironment is hypothesized to create a “sink” that could limit tumor binding of MSLN-targeted anticancer therapies. We describe JNJ-79032421, a bispecific antibody (BsAb) targeting cluster of differentiation (CD) 3 on T cells and the membrane-restricted, non-shed region of full-length MSLN on cancer cells. Crystal structure analysis confirmed JNJ-79032421 binding to the membrane-restricted, non-shed C-terminal region of MSLN. Using Western blotting, enzyme-linked immunosorbent assay (ELISA), fluorescence-activated cell sorting (FACS), and immunohistochemistry (IHC), elevated levels of sMSLN were detected in blood, serosal fluids, and tumor tissue of patients and tended to increase with MSLN tumor expression. Binding and cytotoxicity of JNJ-79032421 against cancer cell lines were unaffected by the presence of sMSLN. In cancer cell lines and cell line–derived and patient-derived xenograft mouse models, increasing levels of MSLN expression led to increased JNJ-79032421 potency. In contrast, MSLNxCD3 BsAbs, binding of which is non–membrane-restricted, demonstrated a loss of cytotoxicity in the presence of sMSLN. This “sink” effect was most pronounced for high-affinity non–membrane-restricted BsAbs. Increases in CD45^+^ and CD8^+^ T-cell infiltration, as well as CD8^+^ T-cell activation, observed in xenograft tumor tissues were time-dependent and JNJ-79032421 dose–dependent. Overall, these data suggest that JNJ-79032421 may offer meaningful clinical activity by avoiding binding to sMSLN.

## Graphical Abstract

**Figure:**
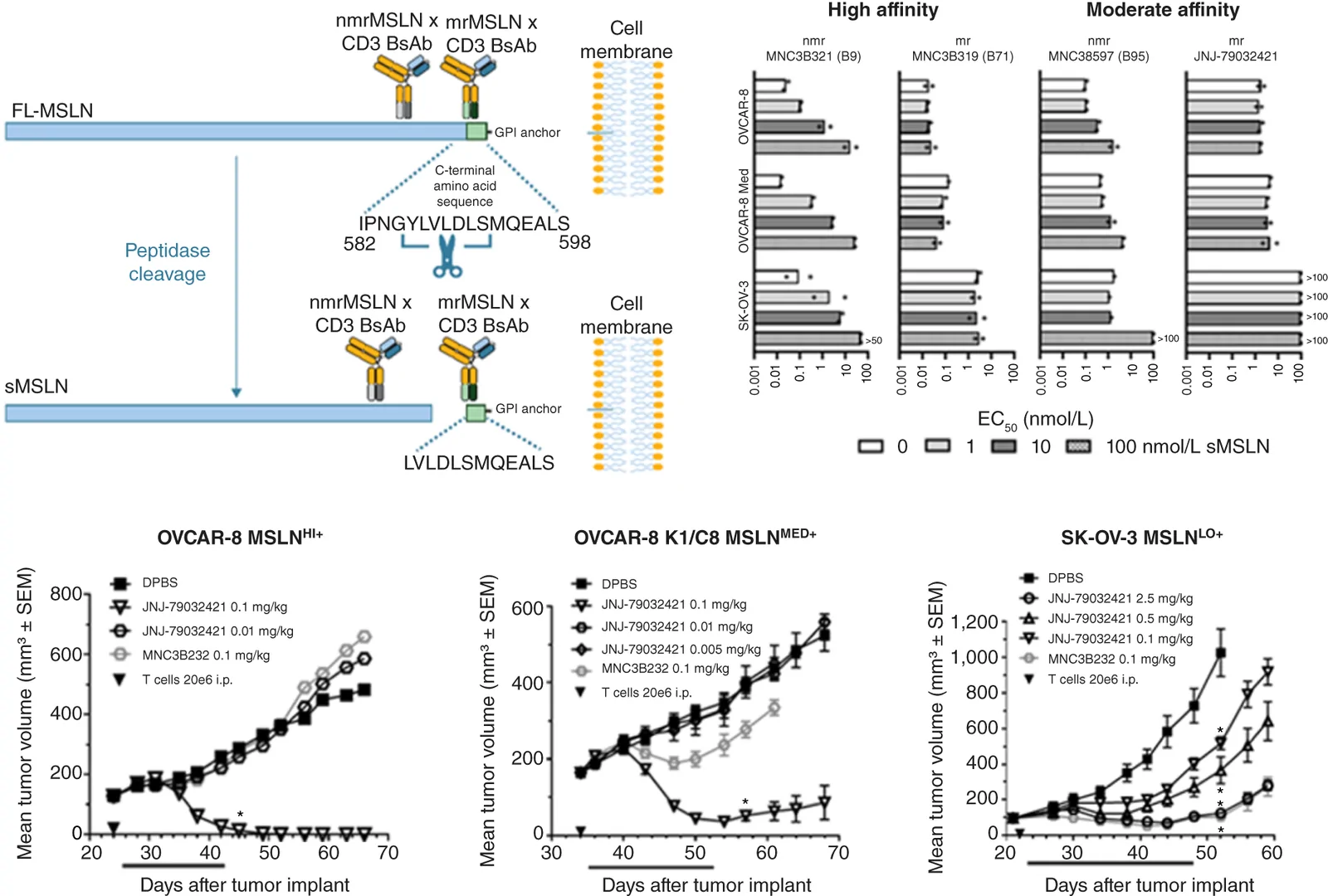

## Introduction

The surface glycoprotein mesothelin (MSLN) is overexpressed in several cancer types, and multiple MSLN-targeting agents have been tested in the clinic, including monoclonal antibodies (mAb), antibody–drug conjugates (ADC), immunotoxins, chimeric antigen receptor T cells (CAR-T), and bispecific antibodies (BsAb; refs. 1, 2). Although MSLN is widely expressed in cancer, the efficacy of targeted agents has been so far limited (3).

MSLN is a lineage-restricted glycosylphosphatidylinositol (GPI)-anchored cell membrane glycoprotein that is overexpressed in many cancers (4). Although discovered in the early 1990s when searching for druggable targets for solid tumors (5), its biological role remains largely unknown, with no measurable phenotype in MSLN-knockout (KO) mice (6). In cancer, there is strong evidence that MSLN plays a role in tumor cell proliferation and likely participates in peritoneal dissemination; however, the mechanisms remain largely elusive (4). MSLN healthy-tissue expression is limited to pleura, pericardium, peritoneum, and some epithelial cells of various tissues, with no expression detected in the parenchyma of vital organs; in tumors, the protein is expressed in most mesotheliomas, pancreatic cancer, and ovarian cancer, as well as in some other cancer types (7–9).

MSLN is cleaved from the cell membrane by the sheddase TNFα-converting enzyme (10, 11) and other enzymes (12) that all cleave near its C-terminus, 7 to 14 amino acids distal from the GPI anchor. Proteolytic cleavage of MSLN releases soluble MSLN and leaves a short stub GPI-anchored to the cell membrane. Large amounts of shed MSLN (sMSLN) in body fluids, and particularly in the tumor microenvironment (13), act as a “sink” to agents targeting MSLN (10). In addition, it has been shown that sMSLN competes with therapeutic antibodies already bound to MSLN-expressing cells, thus neutralizing their activity (14).

This ‘sink’ effect liability has been acknowledged within the field, and novel agents in early clinical development are formulating strategies to address this. Our approach makes use of JNJ-79032421, a BsAb targeting cluster of differentiation (CD) 3 on T cells and membrane-restricted, full-length MSLN on tumor cells, while avoiding binding to sMSLN and loss of activity due to MSLN shedding, an approach that recently has been implemented in anti-MSLN CAR-Ts (15). Here, data are presented demonstrating that JNJ-79032421–mediated T-cell targeting to the membrane-restricted (mr)MSLN region leads to superior preclinical activity in high MSLN-expressing, high sMSLN-shedding tumor models compared with low MSLN-expressing models representing benign MSLN-positive tissues.

## Materials and Methods

### Test agents and controls

Test agents and their affinities are listed in Table 1. All antibodies were prepared in-house; phycoerythrin (PE)-bound MSNB457-PE and MSNB570-PE were sourced from Thermo Fisher Scientific. The CD3 arm was single-chain fragment variable (scFv) W245 LH of medium potency with a T-cell binding 50% effective concentration (EC_50_) of 28.6 to 30.4 nmol/L depending on the T-cell donor (Supplementary Fig. S1). JNJ-79032421 (aka MNC3B304), MSNB457, MSLNB570, MSNB71, and W245 LH sequences and biophysical characteristics are described in WO2024/089551Al. JNJ-79032421 is described in Fig. 17, Fig. 18, Table 15, and example 5; MSNB457 in Table 4; MSLNB570 in Table 8; MSNB71 in Table 1; and CD3 scFv W245 LH in Table 14 (16). The sequences of the heavy-chain (HC) and light-chain variable regions (v-region) of scFv W245, MSNB457, MSLNB570, MSNB71, MSNB95, MSNB90, and MSNB9 are described in Supplementary Table S1 in the Supplementary Materials.

**Table 1.** Test agents and their affinities.

Antibody	Description of the agent	MSLN Fab xCD3 scFvW245 LH KiHBsAb	Fab	Hu/Rb chimeric Ab	MSLN K_D_ (nmol/L)
MSNB457	Human IgG1 antibody binding to mrMSLN (FL-MSLN); parental antibody to JNJ-79032421	JNJ-79032421	MSLNB703	​	4.64 nmol/L
MSNB570	Human IgG1 that binds to mrMSLN (12aa peptide and to FL-MSLN)	MNC3B303	​	​	3.6 nmol/L
MSNB71	Human IgG1 that binds to mrMSLN (12aa peptide and to FL-MSLN)	MNC3B319	​	MNC3B30	0.2 nmol/L
MSNB95	Human IgG1 that binds to nmrMSLN (FL-MSLN and sMSLN)	MNC3B597	​	​	4.0 nmol/L
MSNB90	Human IgG1 that binds to nmrMSLN (FL-MSLN and sMSLN)	​	​	​	0.5 nmol/L
MSNB9	Human IgG1 that binds to nmrMSLN (FL-MSLN and sMSLN)	MNC3B321 and MNC3B232^a^	​	​	0.04 nmol/L

Abbreviations: Ab, antibody; FL, full length; Hu, human; Ig, immunoglobulin; KiH, knob-into-hole; LH, light heavy; Rb, rabbit.

a CD3 scFv W245 LH x MSNB9 Fab BsAb generated using Zymeworks Fc heterodimerization mutations but with identical CD3 and MSNB9 binding arms as MNC3B321. The CD3 scFv W245 LH arm was of medium potency (Supplementary Fig. S1) with T-cell binding EC_50_ of 28.6 to 30.4 nmol/L depending on the T-cell donor.

The BsAb was prepared as follows. JNJ-79032421 was expressed in stably transfected Chinese hamster ovary cells. The fed-batch culture was harvested clarified by centrifugation and filtration. JNJ-79032421 was purified from the culture supernatant using a Protein A column (GE HealthCare). After the supernatant was loaded onto the column, bound protein was eluted with five column volumes of 0.1 mol/L sodium acetate (pH 3.5). Protein fractions were neutralized immediately by the addition of 2.5 mol/L Tris-HCl (pH 7.2) to 15% (v/v) of the elution fraction volume. Peak fractions were pooled and further purified by cation exchange chromatography using CaptoS ImpAct resin (GE HealthCare). The protein was eluted from the column with increasing NaCl gradient in 20 mmol/L 2-(N-morpholino)ethanesulfonic acid pH 6.5. The peak fractions containing monomeric protein were pooled, dialyzed into 10 mmol/L L-histidine (pH 6.5), and then filtered (0.2 μm). The concentration of purified protein was determined by absorbance at 280 nm. The quality of the purified protein was assessed by SDS-PAGE and analytic size-exclusion high-performance liquid chromatography (HPLC; Agilent HPLC system). The endotoxin level was measured using a turbidimetric limulus amebocyte lysate assay (Pyrotell-T, Associates of Cape Cod).

MSLN stub, a 12-aa C-terminal MSLN peptide not cleaved from the cell membrane (residues 587–598; LVLDLSMQEALS), was synthesized by Thermo Fisher Scientific. Full-length MSLN with C-terminal histidine-tag (residues 296–598) and sMSLN (residues 296–591) were prepared in-house. The extracellular domain 17-mer peptide of human MSLN (residues 582–598; IPNGYLVLDLSMQEALS), used for crystallography, was synthesized at Vivitide.

Ascites, pleural fluids, and tumor tissues from patients with ovarian, lung, or pancreatic cancer were obtained for diagnostic purposes, coordinated by BioIVT and Discovery Life Sciences, with informed consent from all subjects.

### Cell lines

An overview of the cell lines used is provided in Supplementary Table S2. All cell lines were maintained at 37°C and 5% CO_2_ in the specified complete culture medium. Cells used for *in vitro* experiments were confirmed to be *Mycoplasma* free using MycoAlert PLUS Mycoplasma Detection Kit (#LT07-710) two times per year. Cell stocks prepared for *in vivo* studies at Johnson & Johnson were authenticated by DNA fingerprinting and short tandem repeat (STR) matching analysis by Analytical Biological Services, Inc. (ABS) and confirmed to be *Mycoplasma* free by polymerase chain reaction (PCR) performed by ABS. The number of passages between defrosting the stock and inoculation was maximum 6. Patient-derived xenograft (PDX) cell stocks from Charles River Laboratories were confirmed to be *Mycoplasma* free by PCR. ATCC and European Collection of Animal Cell Cultures use PCR-based *Mycoplasma* tests and DNA fingerprinting and STR matching analysis for authentication on cell line distribution stocks. Following information is provided in Supplementary Table S2: cell line name, tissue type, commercial source, MSLN expression levels, culture media, sex, acquisition date, *Mycoplasma* and authentication dates, and passage numbers used in experiments.

### *In vivo* studies

Experiments adhered to the Guide for the Care and Use of Laboratory Animals (17) and the Animal Welfare Act and were approved by the local ethics committees of Johnson & Johnson, Spring House, PA [Institutional Animal Care and Use Committee; cell line–derived xenograft (CDX) models], or Charles River Discovery Research Services, Germany (PDX models).

Female nonobese diabetic (NOD)/severe combined immunodeficient (SCID) gamma (NSG) mice (NOD.Cg-*Prkdc*^*scid*^*Il2rg*^tm1Wjl^/SzJ, The Jackson Laboratory, RRID: IMSR_JAX:005557) were subcutaneously implanted with 1 × 10^6^ OVCAR-8 (RRID: CVCL_1629) or OVCAR-8 K1/C8 (OVCAR-8 engineered to express medium levels of MSLN) or 5 × 10^6^ SK-OV-3 (RRID: CVCL_0532) cells. For PDX models, mice received tumor fragments of LXFA 289, PAXF 1657, PRXF-MRI-H 1579, or LXFA 1041. To humanize the immune system, human pan-T cells were activated *in vitro* and expanded using a T-cell expansion kit (Miltenyi Biotec, #130-091-441) and IL2 (Miltenyi Biotec, #130-097-743) supplementation at a concentration of 0.1 µg/µL, starting 3 days after thawing. *In vitro* T-cell expansion continued for an additional 11 to 15 days, for a total of 13 to 17 days in culture. On the day of engraftment into mice, beads were removed from the T cells using magnets, and cells were resuspended in serum-free medium at a concentration of 1 × 10^8^ cells/mL for an intraperitoneal injection of 2 × 10^7^ cells in 0.2 mL per mouse. Mice were given a fragment crystallizable (Fc) block (Bio X Cell, #AB_2736987; 0.2 mg/mouse) and immunoglobulin infusion Gammagard liquid 10% (#NDC 00944-2700-05; 10 mg/mouse) to offset the low immunoglobulin environment. Mice were randomized into groups of 9 to 10 by tumor volume, with treatments initiated via intraperitoneal administration of JNJ-79032421 (0.01, 0.1, 0.5, or 2.5 mg/kg), MNC3B232 (0.1 mg/kg), or Dulbecco’s phosphate-buffered saline (DPBS; no calcium, no magnesium, Sigma/Gibco, D8537/14190-250) twice weekly for six to eight doses. In the PAXF 1657 study, nullxCD3 was used as a control (2.5 mg/kg). For pharmacodynamics, mice (*n* = 33 per treatment with *n* = 3–5 per treatment per time point) received JNJ-79032421 (0.05 or 0.5 mg/kg) or DPBS and tumors were collected 24 hours after the second, third, and fourth doses.

Efficacy data are expressed as individual values or means ± standard error of the mean (SEM) using GraphPad Prism (Dotmatics, SCR_002798). Percent Δ tumor growth inhibition (TGI) was calculated as the difference in mean tumor volumes between treated and control groups relative to day 0. A complete response (CR) is defined as complete tumor regression with no palpable tumor. Statistical analysis was performed using linear mixed-effects analysis for tumor growth slopes or treatment per time point, with significance set at *P* ≤ 0.05.

### Enzyme-linked immunosorbent assay

The LEGEND MAX Human Mesothelin ELISA Kit (BioLegend, #438607) was utilized to assess MSLN levels in serum samples from healthy male (*n* = 60; Seralab) or pregnant female donors (*n* = 31; Seralab) and patients with mesothelioma (*n* = 7), ovarian cancer (*n* = 9), and lung cancer (*n* = 81), as well as from ovarian cancer ascites (*n* = 6) and lung cancer pleural effusions (*n* = 5). The enzyme-linked immunosorbent assay (ELISA) detected sMSLN, secreted MSLN, and mrMSLN in tumor lysates, whereas only secreted and sMSLN were present in culture supernatants and sera. Plates were measured on an EnVision plate reader (PerkinElmer, SCR_018038).

To detect membrane-bound full-length MSLN, a Meso Scale Discovery (MSD)–based ELISA was developed. A standard MSD plate (#L15XA) was coated with capture antibody MSNB71 (in-house, Lot #MSNB71.005) and subsequently incubated with CDX lysate samples. Membrane-bound full-length MSLN was detected with mouse anti-MSLN mAb A88 (Rockland, AB_1057650; directed to MSLN amino terminus) primary and anti–mouse-SULFO-TAG secondary antibodies (MSD, #R32AC) and measured using a MESO SECTOR S 600 plate reader (MSD, model 1201). sMSLN levels were estimated by subtraction of full-length MSLN from total MSLN levels (LEGEND Max ELISA kit, BioLegend, #438607).

### Western blotting

PDX samples from the lung (*n* = 88), pleura (*n* = 9), ovary (*n* = 7), and pancreas (*n* = 48) were analyzed by Western blotting. Protein lysates (5 mg/mL) were obtained from Charles River Laboratories. Lung adenocarcinoma tissues (*n* = 21; Asterand) and OVCAR-8, OVCAR-8 K1/C8, and SK-OV-3 CDX (*n* = 1) were crushed using cryoPREP Automated Dry Pulverizer (Covaris, SCR_026012), according to the manufacturer’s protocol and lysed, with protein concentrations determined using the BCA Protein Determination kit (Pierce, #23225) with albumin standard (Pierce, #23209). Lysates were treated with peptide:N-glycosidase F (Sigma, #P7367) and separated by NuPAGE gel electrophoresis (Invitrogen, #NP0322BOX) at 15 to 20 µg protein per lane after which protein transfer to the polyvinylidene fluoride (PVDF) regular membrane (Blot Gel Transfer Stacks) was done on iBlot2 (Invitrogen, IB401001), following the manufacturer’s protocol. PVDF membranes were blocked and incubated with primary antibodies [Mesothelin (D4X7M) Rabbit mAb, Cell Signaling Technology, AB_2799681, recognizing uncleaved (full-length) and cleaved forms of MSLN, and mouse anti-vinculin (Upstate, #05-386)]. Alexa 680 goat antirabbit antibody (Life Technologies, AB_2535736) and DyLight 800 donkey antimouse antibody (Rockland, AB_1660927) were used to detect the primary antibodies. Membranes were scanned on an Odyssey CLx Imager (LI-COR Biosciences, SCR_014579). Data visualization and analysis were carried out with either Odyssey analysis software or Image Studio Odyssey software (LI-COR, SCR_015795). Quantitation of detected bands was performed using Image Studio Analysis software (version 5.2).

### Surface plasmon resonance

Using a Biacore 8 K surface plasmon resonance (SPR) system, BsAbs were captured on a goat anti–human Fc-specific polyclonal antibody CM4 sensor chip (GE, #BR-1005–34; Jackson ImmunoResearch; #109-005-098) at 15 µg/mL. Full-length recombinant MSLN with a 6× His tag was injected in Single-Cycle Kinetics mode at concentrations ranging from 3.7 to 100 nmol/L with 180 seconds association time and 600 seconds dissociation time (*n* = 1 measured per sample). Raw binding data were processed using double referencing and then analyzed using a 1:1 simple Langmuir binding model.

### MSLNB703–MSLN crystallization, data processing, and structure determination

The 17-mer peptide of human MSLN (residues 582–598) and fragment antigen-binding (Fab) MSLNB703 were mixed at a 2:1 mol/L ratio for 2 hours on ice. The Fab–peptide complex was crystallized with a Mosquito LCP (SPT Labtech) crystallization robot (SCR_020864) at 20°C using the sitting drop vapor-diffusion method from solution containing 30% polyethylene glycol (PEG)1500 (Hampton Research; #HR2-525). Crystals belonging to the triclinic space group P1 and with unit cell dimensions of 60.2 to 79.7 × 86.8 Å contained four Fab–peptide complexes in the asymmetric unit, each with a single-bound peptide. Crystals of the Fab–peptide complex were harvested in cryoprotectant [30% PEG1500 and 30% (volume per volume, v/v) glycerol (Hampton Research, #HR2-623)] and flash-frozen in liquid nitrogen. X-ray diffraction data for the Fab–peptide complex were collected at −180°C using station 17-ID (IMCA-CAT). Data were processed using autoPROC (SCR_015748; ref. 18) and STARANISO (Global Phasing, SCR_018362), and the resolution cutoff for the highest resolution shell used the following criteria: CC(1/2) ≥0.3, R(pim) ≤0.6, and local I/sigI ≥1.2. Phenix (SCR_014224) was employed for phasing and refinement, and model building was performed using Coot (SCR_014222; refs. 19, 20). The structure of the Fab–peptide complex was determined with anisotropic resolution cutoffs at 1.96, 2.03, and 2.32 Å by molecular replacement using Phaser (SCR_014219; ref. 21), and a homology model of the Fab as a search model was generated using MOE (Chemical Computing Group ULC, SCR_014882). All structures were refined using individual B-factor refinement on Fab and peptide atoms, and local noncrystallographic symmetry restraints were automatically applied. The Ramachandran plot of the final model calculated using MolProbity (SCR_014226; ref. 22) showed 97.38% of all residues in the favored region, 2.62% in the allowed region, and no outliers.

### T-cell redirection assays

Cells [i.e., OVCAR-8, OVCAR-8 NucLightRed, OVCAR-8 LM2/B6, OVCAR-8 K1/C8, OVCAR-8 MSLN-KO/recombinant GPI-12aa C-term MSLN stub (OVCAR-8 GPI-12aa C-term), NCI-H838, COR-L23, Capan-2, Capan-2 NucLightRed, NCI-H292, NCI-H292 NucLightRed, MeT-5A, LXFA 983L, MDA-MB-231, SK-OV-3, HNXF 1859L, OVXF 1023L, and MSTO-211H] were detached (Enzyme-Free Cell Dissociation buffer, Thermo Fisher Scientific, #13151014), collected, washed in DPBS, and resuspended in assay medium [Roswell Park Memorial Institute (RPMI) 1640 without phenol red (Gibco, #32404014), 2 mmol/L L-glutamine (Sigma, #G7513), 10% fetal bovine serum (FBS; Biowest, #S1810-500), 1 mmol/L sodium pyruvate (Sigma, #S8636), and 0.1 mmol/L nonessential amino acids (Sigma, #M7145)] and 1 × 10^4^ cells/well incubated overnight at 37°C and 5% CO_2_.

To determine the effect of MSLN expression levels on T cell–mediated cytotoxicity, cytotoxicity assays were performed for each cell line with JNJ-79032421 and MNC3B232 across a concentration range of 100 nmol/L to 0:01 pmol/L (fivefold serial dilutions).

To measure the impact of sMSLN, MNC3B321, MNC3B319, MNC3B597, and JNJ-79032421 MSLNxCD3 BsAbs (final concentration range: 100 nmol/L to 0:01 pmol/L, fivefold dilutions), T cell–induced cytotoxicity assays were performed in high MSLN-expressing (MSLN^HI+^) OVCAR-8, medium MSLN-expressing (MSLN^MED+^) OVCAR-8 K1/C8, and low MSLN-expressing (MSLN^LO+^) SK-OV-3 cells in the presence of sMSLN (0, 1, 10, or 100 nmol/L), using T cells from healthy donors at a 1:1 effector-to-target (E:T) ratio. Antibodies (fivefold dilutions in assay medium) were mixed with sMSLN, and the mixture was added to the plate with cells. Next, T-cell suspensions (1 × 10^4^ or 3.33 × 10^3^ T cells in assay medium, for a 1:1 or 0.3:1 E:T ratio) were added to the cells, followed by the addition of Caspase 3/7 Green reagent (Essen BioScience, #4440) or Annexin V Green reagent (Essen BioScience, #4642). The plate was incubated at 37°C and 5% CO_2_ in Incucyte S3 (Sartorius, SCR_023147), and cell growth and Caspase 3/7 Green signal were recorded every 4 hours for 5 days.

Cytotoxicity experiments to determine the impact of MSLN expression levels on potency were performed between two and six times per cell line and per E:T ratio. Individual log EC_50_ values were plotted against log of receptor density values. Potency and receptor density were analyzed for association using linear regression, with time, E:T ratio, and donor covariates tested but excluded for insignificance from the final model.

The sMSLN competition experiment was performed twice, and geometric mean (geomean) EC_50_ values were calculated. Fold changes (FC) were calculated from geomean EC_50_ values obtained in the presence of 1, 10, or 100 nmol/L sMSLN versus the geomean EC_50_ without sMSLN.

### Binding competition assay

Binding of MSNB457 (mrMSLN mAb) or MSNB90 [non–membrane-restricted (nmr)MSLN mAb] to OVCAR-8 cells was assessed in the presence or absence of MSLN. MSNB457 and MSNB90 (final concentrations: 0.011–33 nmol/L, fivefold dilutions) were preincubated for 30 minutes with or without 1 µmol/L MSLN peptide, full-length MSLN, or sMSLN and then tested for binding to OVCAR-8 cells on a BD FACSVerse flow cytometer (BD Biosciences, #651155). The median fluorescence intensity (MFI) of test antibody binding was divided by MFI of control antibody binding to calculate FC over control.

### Assessment of MSLN receptor density on normal and tumor primary cells

Primary cells in suspension were collected from ascites and pleural effusions from patients with ovarian, pancreatic, and lung cancers. Serous effusions were filtered through a 100-µm nylon cell strainer (Falcon, #352360) to obtain single cells and separated from cleared supernatant by centrifugation. Cells were treated with red blood cell (RBC) lysis buffer (Lonza, #BP10-548E) to remove RBCs, counted, and suspended in primary cell medium [RPMI 1640 medium containing 1 mmol/L sodium pyruvate, 4.5 g/L glucose (Sigma, #G8769), 0.75 g/L sodium bicarbonate (Sigma, #S8761), 2 mmol/L glutamine (Sigma, G7513), 77 USP units/L human recombinant insulin (Gibco, #12585-014), 1% penicillin/streptomycin (Sigma, #P4458), 570 µg/L amphotericin B (Sigma, #A2942), and 10% autologous cleared serous fluid]. After incubation (1 hour, 37°C), viability was assessed with LIVE/DEAD Fixable Near-IR Dead Cell Stain (Thermo Fisher Scientific, #L10119). Next, after Fc blocking, stain cocktail [i.e., CD45 clone H130, BV510 (BD Biosciences, #563204), diluted 1:40; CD33 clone P67-6, BB700 (BD Biosciences, #745834), diluted 1:160; CD71 clone M-A712, BV786 (BD Biosciences, #563768), diluted 1:40; CD326/epithelial cell adhesion molecule (EpCAM) clone EBA-1, BV421 (BD Biosciences, #563180), diluted 1:50; podoplanin clone LpMab-17, conjugated to BUV395 (BD Biosciences, #747630), diluted 1:20; CD141/thrombomodulin clone 1A4, BB515 (BD Biosciences, #565084), diluted 1:50; and MSNB570-PE in BD Brilliant stain buffer (BD Biosciences, #659611)] was added, and different cell populations were separated by fluorescence-activated cell sorting (FACS) on LSRFortessa (BD Biosciences, SCR_018655). MSNB570-PE was used to detect MSLN. Cells of nonhematopoietic lineage were gated out in the CD45 versus CD33 plot. The mesothelial cells and the cancer cells were part of the CD45^−^CD33^−^ cell population, and in most cases these cells were CD71^+^. EpCAM-positive cells were identified as epithelial cancer cells and podoplanin-positive (and thrombomodulin-positive) cells as mesothelial cells. The geomean for PE on these final populations served as a measure for MSNB570-PE binding and was used for further analysis and antibody binding capacity (ABC) calculations. ABC for MSLN was assessed using Quantum Simply Cellular beads (Bangs Laboratories, anti-human, #816A); events were collected for analysis on LSRFortessa.

### MSLN, cytokeratin, and CD8 immunohistochemistry

Formalin-fixed, paraffin-embedded (FFPE) tumor samples were sectioned at 4 µm and placed on positively charged glass slides. Before immunostaining, paraffin sections were deparaffinized followed by antigen retrieval using a heat-induced epitope retrieval method. All sections were treated with 4% H_2_O_2_ to block endogenous peroxidase and some sections (MNC3B30.001 and pancytokeratin) with Dako Protein Block (Agilent, #X0909) to block endogenous protein. The sections were then incubated at room temperature with MSLN [clone MNC3B30.001; diluted 0.4 µg/mL; incubated 60 minutes using the Leica Bond Rx (SCR_025548)], MSLN (clone 5B2; Leica Biosystems, #PA0373, AB_563875; pre-diluted; incubated 15 minutes using the Leica Bond Rx), pancytokeratin (clone AE1/AE3+8/18; Biocare Medical, #CM162C, AB_10581786; diluted 1:100; incubated for 15 minutes using the Leica Bond Rx), or CD8 [clone SP57; Roche Diagnostics Ventana, #790-4460, AB_2335985; prediluted; incubated for 60 minutes using Ventana Discovery Ultra (SCR_021254)]. Subsequently, the sections were incubated with horseradish peroxidase polymer (BioLegend, #405103) and 3,3′-diaminobenzidine substrate to detect the primary antibody. Finally, hematoxylin was applied to counterstain the sections. Representative micrographs of tumors were taken at 40× magnification. Controls and samples were reviewed by a Johnson & Johnson pathologist.

### Assessment of tumor-infiltrating lymphocytes *ex vivo* by flow cytometry

Tumors were excised, minced, and dissociated into single-cell suspensions with the Tumor Dissociation Kit for murine tissues (Miltenyi Biotec, SCR_020285) according to the manufacturer’s instructions using gentleMACS Octo Dissociator with Heaters (Miltenyi Biotec, SCR_020271). Samples were filtered through 70-µm cell strainers and lysed for RBCs using ACK lysis buffer (Gibco, #A1049201). Samples were plated at 2 × 10^7^ viable cells/well and stained with Fc block and LIVE/DEAD BV480 (Thermo Fisher Scientific, #L34957). Antibody cocktails were assembled with the following stains: hCD8-BUV496 (clone RPA-T8; BD Biosciences, AB_2916884), hCD69-BUV395 (clone FN50; BD Biosciences, AB_2738770), hKi67-BV711 (clone B56; BD Biosciences, AB_2738406), and hCD45-BV480 (clone HI30; BD Biosciences, AB_2739517). Flow cytometry data were collected on BD LSRFortessa instruments and analyzed using FlowJo software (Becton Dickinson, SCR_008520, version 10).

## Results

### sMSLN quantification in bodily fluids and tumor tissues

**Figure 1.**
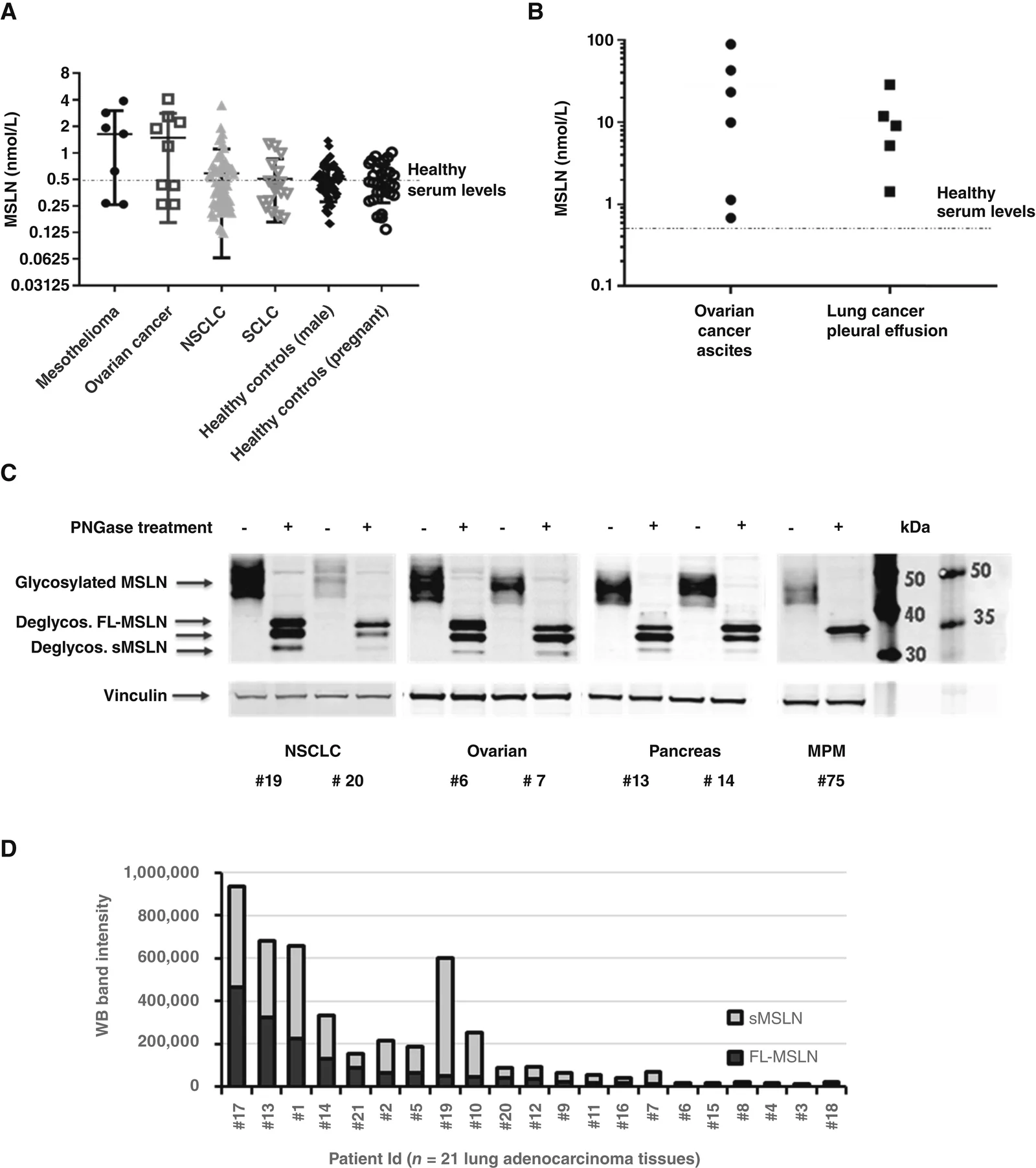
Quantification of (**A**) sMSLN in healthy and cancer sera, (**B**) sMSLN in serosal fluids, (**C**) full-length MSLN and sMSLN in CDXs and PDXs, and (**D**) full-length MSLN and sMSLN in NSCLC adenocarcinoma tumors. sMSLN levels were evaluated by ELISA in (**A**) serum samples from patients with cancer and healthy controls and (**B**) ovarian cancer ascites and lung cancer pleural effusions from patients with cancer. Means (±SD) are graphed; dashed horizontal lines denote the level of sMSLN in healthy-donor serum. **C,** MSLN levels in PDXs were determined by Western blot analysis using a rabbit anti-human MSLN antibody detecting both FL-MSLN and sMSLN. Samples were treated without or with PNGase, enabling SDS-PAGE resolution between FL-MSLN and sMSLN. Representative samples shown. Respective order of PDX models on blot: LXFA 1041 (#19), LXF 1125 (#20), OVXF 1320 (#6), OVXF 1993 (#7), PAXF 1994 (#13), PAXF 1997 (#14), and PXF 1118 (#75). **D,** FL-MSLN and sMSLN in 21 NSCLC adenocarcinoma tissues were quantified by Western blot analysis using a rabbit anti-human MSLN antibody detecting both FL-MSLN and sMSLN. Deglycos., deglycosylated; FL-MSLN, full-length mesothelin; Id, identification; MPM, malignant pleural mesothelioma; NSCLC, non–small cell lung cancer; SCLC, small cell lung cancer; WB, Western blot.

**Figure 2.**
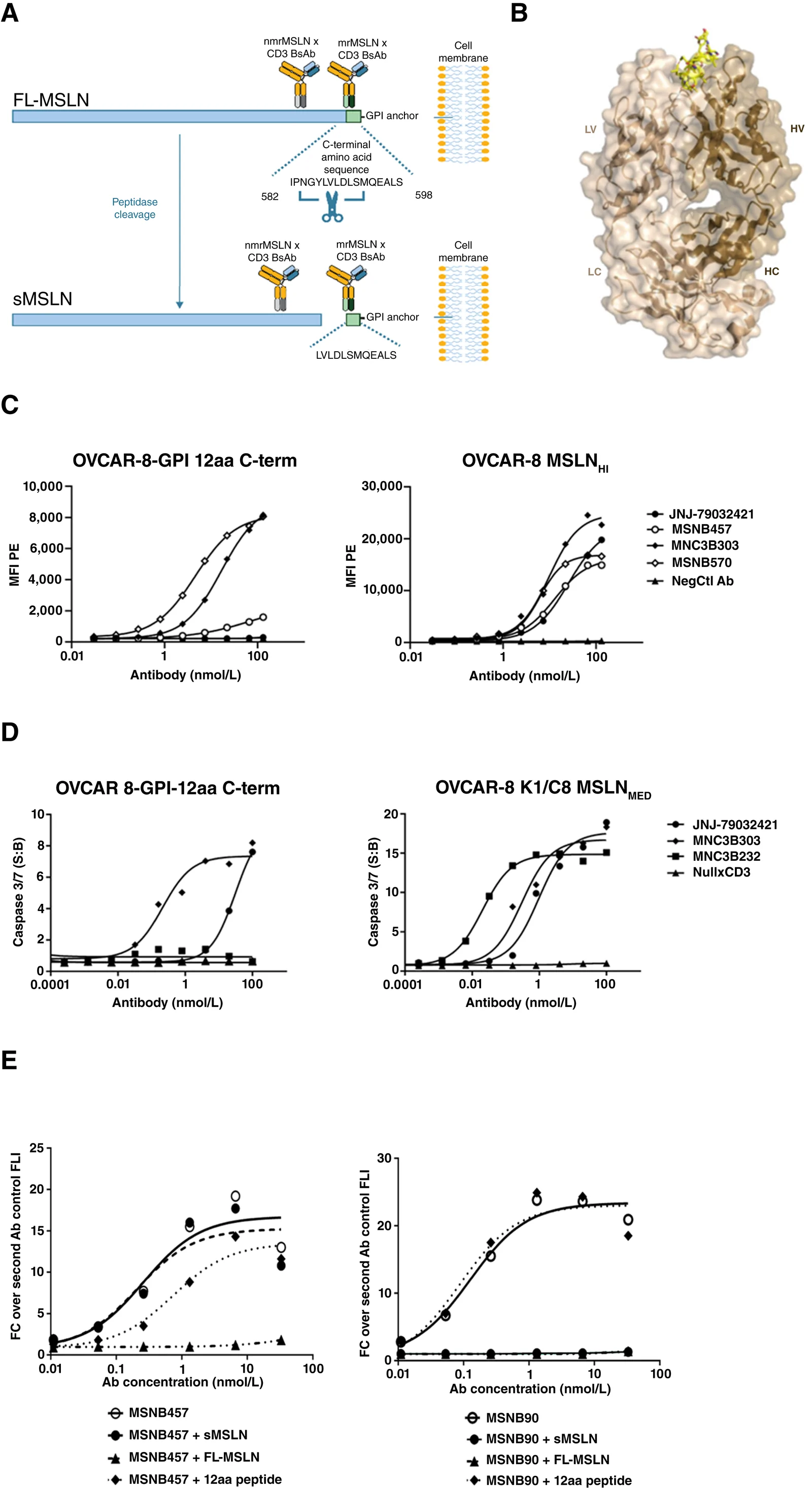
**A,** Cartoon showing antibody binding to full-length MSLN and sMSLN for mrMSLNxCD3 and nmrMSLNxCD3 BsAbs; **B–E,** MSLN binding epitope characterization of JNJ-79032421 and related mr and nmrMSLNxCD3 antibodies. **A,** Graphical representation of the binding regions for mrMSLNxCD3 and nmrMSLNxCD3 BsAbs used. **B,** Binding to OVCAR-8-GPI 12aa C-term and OVCAR-8 MSLN^HI^ expressing cells using MSNB457, MSNB570, and NegCtl mAbs and JNJ-79032421, MNC3B303, and nullxCD3 BsAbs. **C,** T cell–mediated cytotoxicity to OVCAR-8-GPI 12aa C-term and OVCAR-8 MSLN^MED^ expressing cells, with JNJ-79032421, MNC3B303, MNC3B232, and nullxCD3 BsAbs. Studies were conducted using T cells from a healthy donor at a 1:1 E:T ratio. Cytotoxicity displayed after 72-hour incubation. **D,** 2.0-Å crystal structure of MSLNB703 Fab bound to the C-terminal peptide of human MSLN. A molecular surface of MSLNB703 is overlaid on a cartoon representation of the LV and LC domains (colored wheat) and the HV and HC domains (colored dark brown). The MSLN peptide (MSLN aa 582-598, IPNGYLVLDLSMQEALS) is represented as yellow-colored sticks. **E,** Binding of MSNB457 (mrMSLN Ab) or MSNB90 (nmrMSLN Ab) to OVCAR-8 cells in the presence or absence of MSLN proteins. sMSLN lacks 7–amino acid SMQEALS that are in the mrMSLN region; 12–amino acid MSLN peptide LVLDLSMQEALS includes the mrMSLN C-terminal peptide bound by MSNB457. aa, amino acid; Ab, antibody; C-term, C-terminal; FL-MSLN, full-length mesothelin; FLI, fluorescent intensity; HC, heavy constant; HV, heavy variable; LC, light constant; LV, light variable; NegCtl Ab, negative control antibody; S:B, signal over background; WB, Western blot.

Measurement of MSLN in blood and serosal fluids confirmed elevated levels of sMSLN in blood from patients with mesothelioma or ovarian cancer (Fig. 1A). Additionally, serosal fluids from patients with cancer in which normal mesothelial cells and circulating cancer cells can be detected contained up to 179-fold more sMSLN compared with serum from healthy individuals (Fig. 1B). Western blot analysis revealed elevated levels of sMSLN in tumor tissues, often surpassing the amounts of membrane-bound MSLN. Furthermore, the levels of sMSLN generally correlated with increasing levels of membrane-bound MSLN (Fig. 1C and D). Determination of sMSLN in CDX lysates further confirmed that sMSLN concentrations in the tumor microenvironment increased with membrane-bound MSLN expression levels: 0.04 nmol/L in MSLN^LO+^ SK-OV-3, 0.2 nmol/L in MSLN^MED+^ OVCAR-8 K1/C8, and 3.1 nmol/L in MSLN^HI+^ OVCAR-8 CDX lysates. The concentration of sMSLN may be higher at the cell membrane, depending on the tumor density and extent of vascularization. These data suggest that an nmrMSLN-binding antibody, targeting both membrane-bound and sMSLN, may have significantly reduced binding to MSLN-expressing cells because of sMSLN competition in the tumor microenvironment of medium- to high-expressing tumors, whereas mrMSLN-binding antibodies would not suffer from this “sink” effect (Fig. 2A).

### Structure and JNJ-79032421 binding epitope determination

JNJ-79032421 is a BsAb comprising a CD3ɛ-binding scFv arm (CD3W245) and mrMSLN-binding Fab arm (MSNB457). The anti-CD3ɛ v-region was generated by immunizing human antibody transgenic mice (Ablexis) with recombinant CD3ɛ protein, followed by light-chain humanization. The anti-MSLN v-region was also generated by immunizing transgenic mice (Ablexis) with a mouse/human chimeric recombinant MSLN protein to direct the antibody response toward the 52 most C-terminal amino acids of MSLN. JNJ-79032421 was generated by co-expression of the anti-CD3ɛ scFv-Fc HC and the anti-MSLN Fab HC and light chain. The two HCs contain “knob” (T366W) and “hole” mutations (T366S/L368A/Y407V), respectively, to facilitate heterodimerization. Additional mutations (H435R/Y436F) were incorporated into the anti-MSLN Fab HC to disrupt protein A binding and facilitate purification. Mutations to reduce Fc effector functions were also incorporated into both HCs (23).

The binding epitope of JNJ-79032421 was mapped by determining the co-crystal structure of the Fab-binding domain from JNJ-79032421 with a peptide (IPNGYLVLDLSMQEALS) comprising the nonshed region of MSLN (Fig. 2B). The 2.0-Å crystal structure showed seven contiguous MSLN residues, from Leu587 through Met593, interacting with JNJ-7903221 anti-MSLN Fab (Fig. S2). The JNJ-79032421–binding epitope was functionally confirmed by mutating the MSLN residues in this region (Leu589, Asp590, and Met593) to alanine, which eliminated antibody binding. Measured using SPR, JNJ-79032421 demonstrated a binding affinity (K_D_) of 4.64 nmol/L against full-length MSLN, whereas its affinity to sMSLN, which lacks the seven C-terminal MSLN amino acids (SMQEALS), was not measurable. JNJ-79032421 did not bind to and induced poor T cell–mediated cytotoxicity (EC_50_ ∼20 nmol/L) in OVCAR-8 cells engineered to only express the GPI-12aa C-terminus of MSLN, whereas mrMSLN mAb MSNB570 and its MSLNxCD3 bispecific counterpart MNC3B303, respectively, bound to and killed GPI-12aa–expressing cells 100-fold more efficiently (Fig. 2C and D). Close examination of the crystal structure suggests that the lack of JNJ-79032421 potency against GPI-12aa–expressing cells may result from the absence of a critical interaction involving the 14th amino acid from the C-terminus, Gly585 (Fig. 2B; Supplementary Fig. S2). A van der Waals interaction between the aliphatic region of Arg56 (JNJ-79032421) and Gly585 (MSLN) may help position the guanidinium group of Arg56 so that it forms a direct hydrogen bond to the backbone carbonyl oxygen of Leu587.

JNJ-79032421 and MNC3B303 both induced nearly identical, potent T cell–mediated killing in full-length MSLN-expressing OVCAR-8 K1/C8 cells (Fig. 2C and D), which may indicate that cytotoxicity against MSLN-expressing cells is predominantly through targeting of full-length MSLN. Therefore, we prioritized JNJ-79032421 over other mrMSLNxCD3 BsAbs based on its favorable biophysical characteristics.

### Influence of sMSLN on JNJ-79032421 cell binding and T cell–mediated cytotoxicity

Competition experiments with recombinant sMSLN and full-length MSLN using the parental anti-mrMSLN mAb MSNB457 further confirmed the X-ray data showing that the MSLN Fab arm in JNJ-79032421 does not bind sMSLN (Fig. 2E). sMSLN, which lacks the SMQEALS C-terminus at 1 µmol/L, did not displace MSNB457 binding to MSLN-positive cells. However, binding was fully competed by 1 µmol/L recombinant full-length MSLN and was reduced in the presence of 1 µmol/L of the C-terminal 12aa peptide representing the membrane-restricted stub region. The observed incomplete competition by the 12aa peptide is consistent with SPR analysis showing weak binding with a fast on and off rate. By comparison, MSNB90, an nmrMSLN-binding control antibody (K_D_ = 0.4 nmol/L) that binds outside the stub region, was competed by both full-length MSLN and sMSLN but not by the C-terminal 12aa peptide.

**Figure 3.**
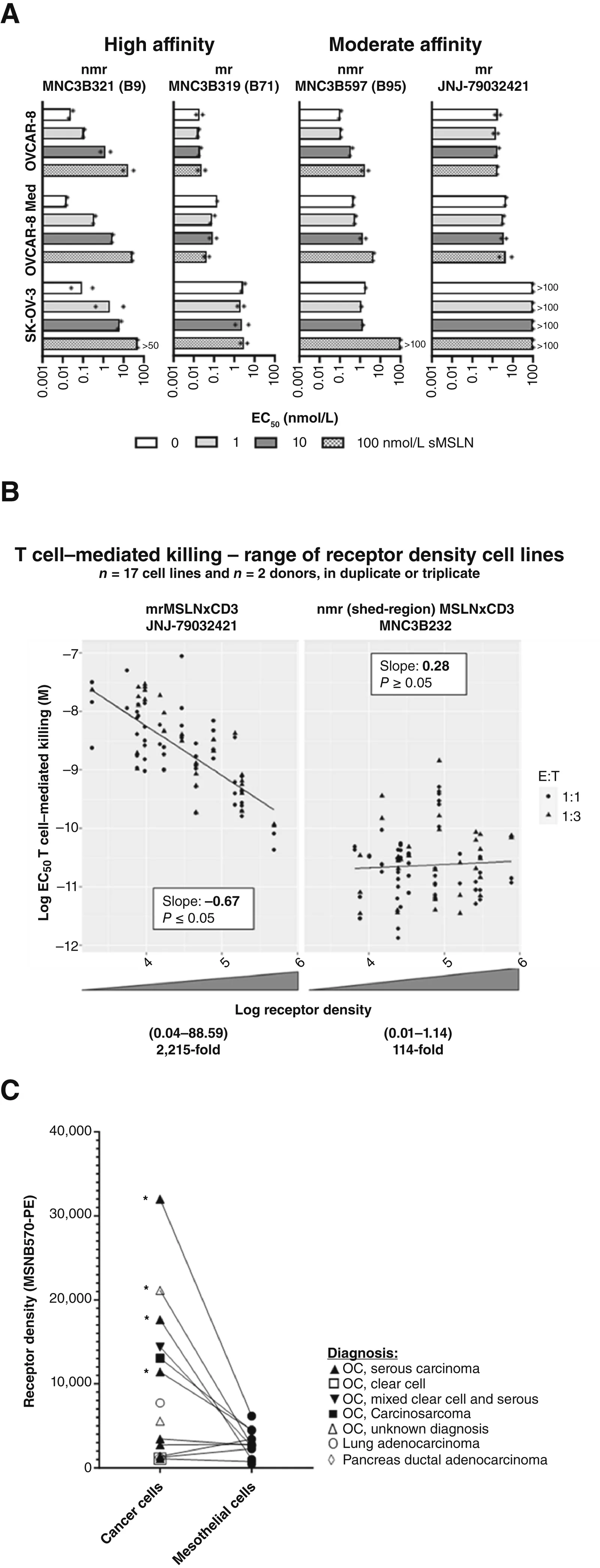
Effects of (**A**) sMSLN and (**B**) MSLN receptor densities on cytotoxicity of anti-mrMSLN (JNJ-79032421) and anti-nmrMSLN BsAbs. **C,** MSLN receptor density on normal and tumor primary cells. **A,** High- [MNC3B321 (nmr) and MNC3B319 (mr)] and low-affinity [MNC3B597 (nmr) and JNJ-79032421 (mr)] MSLNxCD3 BsAb T cell–induced cytotoxicity (EC_50_) against OVCAR-8 (MSLN^HI^), OVCAR-8 K1/C8 (MSLN^MED^), and SK-OV-3 (MSLN^LO^) cells in the presence of sMSLN (0, 1, 10, or 100 nmol/L). Cytotoxicity assays were performed using T cells from healthy donors at a 1:1 E:T ratio. Results were plotted after 72-hour incubation for OVCAR-8 and OVCAR-8 K1/C8; due to reduced signal at 72 hours, results were plotted at 48 hours for SK-OV-3. Single wells per condition were tested. Bars represent the geomean of two individual experiments. Symbol indicates individual EC_50_ values. **B,** T cell–mediated cytotoxicity against a panel of 17 cell lines with different MSLN receptor densities. JNJ-79032421 bound to mrMSLN; nmrMSLNxCD3 MNC3B232 bound to MSLN in a non–membrane-restricted manner. Studies were conducted at 1:1 and 1:3 E:T ratios. Cytotoxicity is displayed after 72-hour incubation. JNJ-79032421 EC_50_ values were graphed relative to MSNB457-PE–derived ABC values. MNC3B232 EC_50_ values were graphed relative to MSNB90-PE–derived ABC values. JNJ-79032421 *P* < 0.05 and MNC3B232 *P* = 0.589. **C,** Cancer cells and benign mesothelial cells isolated from malignant ascites and pleural effusions of patients diagnosed with lung, ovarian, or pancreatic cancer (*n* = 15) were assessed for levels of MSLN expression. MSLN expression (ABC) was determined using MSNB570-PE, which binds mrMSLN. *Indicates an average of two experiments; all other samples were tested once. OC, ovarian cancer.

Likewise, *in vitro* cytotoxicity experiments against SK-OV-3, OVCAR-8 K1/C8, and OVCAR-8 cell lines in the presence of 1 to 100 nmol/L sMSLN confirmed that T cell–induced cytotoxicity with mrMSLNxCD3 BsAbs JNJ-79032421 (K_D_ = 4.64 nmol/L) and MNC3B319 (K_D_ = 0.2 nmol/L) was unaffected in the presence of up to 100 nmol/L sMSLN, whereas T cell–induced cytotoxicity with nmrMSLNxCD3 BsAbs MNC3B597 (K_D_ = 4.0 nmol/L) and MNC3B321 (MSLN K_D_ = 0.04 nmol/L) was reduced up to 14.6- and 283-fold, respectively, in MSLN^HI+^ cells (Fig. 3A; Table 2; Supplementary Table S3). These results demonstrate that JNJ-79032421 binding and T cell–mediated cytotoxicity are not affected by sMSLN, implying that activity is optimally maintained for this membrane-restricted binding approach.

**Table 2.** Geomean EC_50_ values (nmol/L, *n* = 2 experiments) for high- [MNC3B321 (nmr) and MNC3B319 (mr)] and low-affinity [MNC3B597 (nmr) and JNJ-79032421 (mr)] MSLNxCD3 BsAbs T cell–induced cytotoxicity against OVCAR-8 (MSLN^HI+^), OVCAR-8 K1/C8 (MSLN^MED+^), and SK-OV-3 (MSLN^LO+^) cells in the presence of sMSLN.

	sMSLN (nmol/L)											
	0	1	10	100	0	1	10	100	0	1	10	100
	OVCAR-8 (72 hours)				OVCAR-8 K1/C8 (72 hours)				SK-OV-3 (48 hours)			
MNC3B321 (B9)	0.024	0.083	0.560	6.801	0.017	0.255	2.246	23.760	0.089	2.037	6.229	>50
MNC3B319 (B71)	0.008	0.008	0.012	0.008	0.065	0.043	0.037	0.030	2.722	2.104	2.389	2.986
MNC3B597 (B95)	0.066	0.067	0.170	0.966	0.292	0.257	0.818	4.293	1.897	1.167	1.409	>100
JNJ-79032421 (B457)	0.792	0.817	0.970	0.858	2.445	2.313	2.452	1.993	>100	>100	>100	>100

Values represent 72-hour cytotoxicity for OVCAR-8 and OVCAR-8 K1/C8. Values for SK-OV-3 are from 48-hour cytotoxicity time point due to poor curve fit at 72 hours.

### Influence of MSLN expression levels on JNJ-79032421–induced T cell–mediated cytotoxicity

To investigate the extent of T cell–mediated cytotoxicity induced by JNJ-79032421 in cells with different MSLN expression levels, 17 cell lines with receptor densities ranging from 1,700 to 490,000 receptors/cell were selected (Supplementary Table S2). JNJ-79032421–induced (MSLN K_D_ = 4.64 nmol/L) T cell–mediated cytotoxicity was compared with that of nmrMSLNxCD3 BsAb MNC3B232 (MSLN K_D_ = 0.05 nmol/L; Fig. 3B). JNJ-79032421 induced T cell–mediated cytotoxicity in all cells tested with EC_50_ values ranging from 0.04 to 88.59 nmol/L. For JNJ-79032421, T cell–mediated cytotoxicity significantly correlated with MSLN receptor densities, with enhanced cytotoxicity observed against cells with higher MSLN expression levels. In contrast, EC_50_ values of MNC3B232 did not correlate with MSLN expression levels (0.01–1.14 nmol/L), with lower-than-expected cytotoxicity measured in high MSLN-expressing, high MSLN-shedding cancer cells. In this set of experiments, a comparison was not made between mrMSLN- and nmrMSLN-targeting MSLNxCD3 BsAbs with similar affinity to MSLN.

### Xenograft model selection based on MSLN expression levels

**Figure 4.**
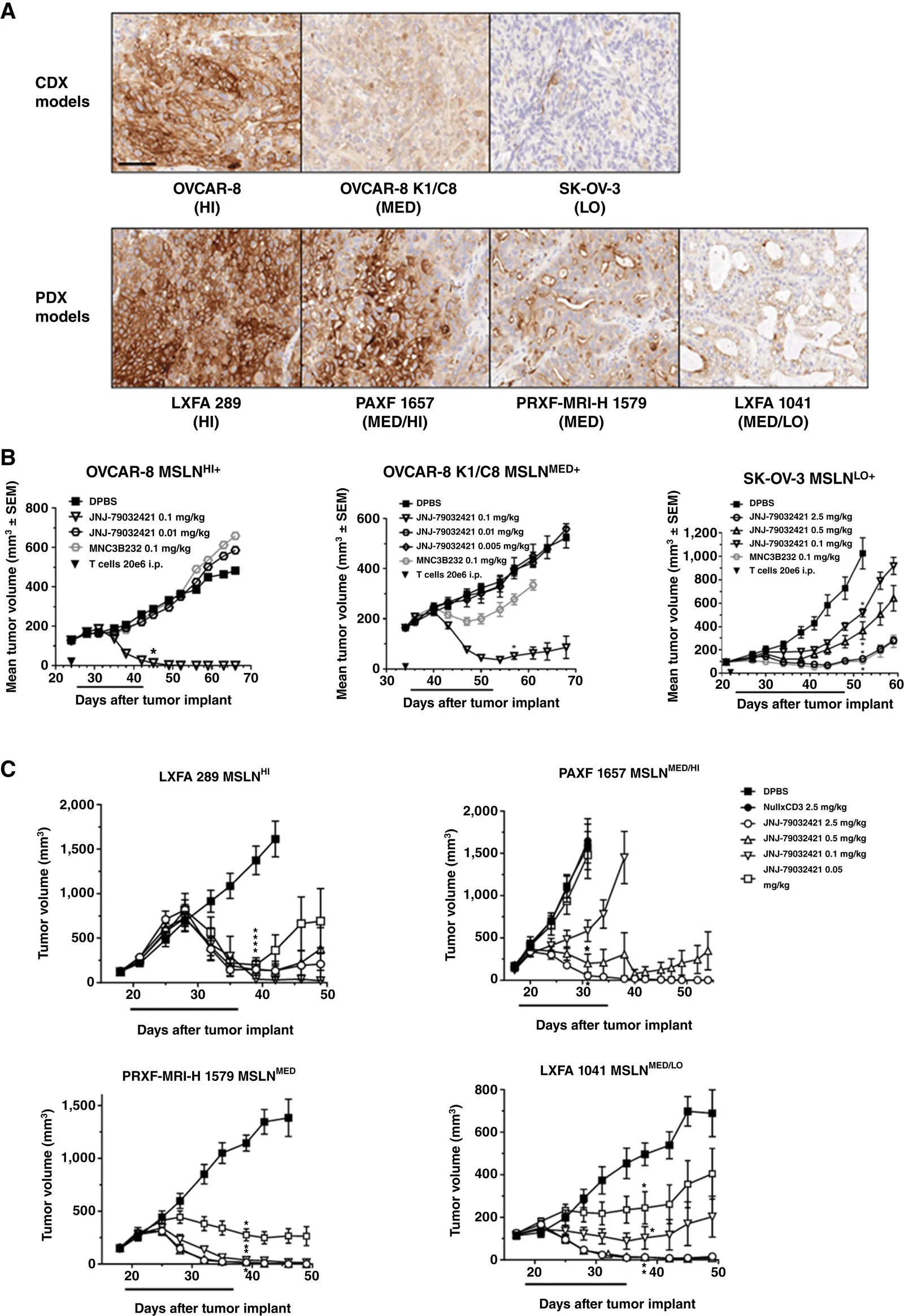
JNJ-79032421 efficacy in xenograft models expressing low to high levels of MSLN. **A,** IHC was performed on xenograft tumors from CDX and PDX models [LXFA 289 (NSCLC), PAXF 1657 (pancreatic cancer), PRXF-MRI-H-1579 (prostate cancer), and LXFA 1041 (NSCLC)] with anti-MSLN antibody MNC3B30, which binds to the membrane-restricted region of MSLN and detects both cleaved C-terminus and full-length MSLN, but not sMSLN. Qualitative MSLN level (HI/MED/LO) assigned below the tumor model in parenthesis. Blue, cell nuclei (hematoxylin); brown, MSLN; magnification bar = 100 µm. **B,** Mean tumor volumes ±SEM over time in CDX models. Tumor cells were implanted on day 0. Mice were humanized with T cells (as indicated by down triangle) on the day of randomization, and treatment was initiated 1 day after T-cell implantation. For OVCAR-8 and OVCAR-8 K1/C8 studies, mice were treated intraperitoneally twice weekly for 3 weeks with JNJ-79032421 at 0.1 and 0.01 mg/kg and MNC3B232 at 0.1 mg/kg. For SK-OV-3, mice were treated intraperitoneally twice weekly for 4 weeks with 0.1, 0.5, and 2.5 mg/kg JNJ-79032421 and 0.1 mg/kg MNC3B232. The dosing period is indicated by the bar below the X-axis. *Denotes significant difference (*P* ≤ 0.05) of the JNJ-79032421 or MNC3B232 treatment group compared with the control (DPBS) group. **C,** Mean tumor volumes ±SEM over time upon treatment of JNJ-79032421 in PDX mouse models. Tumor cells were implanted on day 0. Mice were humanized with T cells (as indicated by down green triangle) on the day of randomization, and treatment was initiated 1 day after T-cell implantation. Mice were treated intraperitoneally twice weekly for 3 weeks on days 19, 22, 26, 29, 33, and 36 (LXFA 289 MSLN^HI+^ and PRXF-MRI-H 1579 MSLN^MED+^) and on days 18, 21, 25, 28, 32, and 35 (PAXF 1657 MSLN^MED+/HI+^ and LXFA 1041 MSLN^MED+/LO+^). The dosing period is indicated by the bar below the X-axis. *Denotes significant difference (*P* ≤ 0.05) of JNJ-79032421 treatment groups compared with the DPBS group (LXFA 289, PRXF-MRI-H 1579, and LXFA 1041) or between groups treated with nullxCD3 and JNJ-79032421 (PAXF 1657). JNJ-79032421 at 0.1 mg/kg resulted in >100% ΔTGI of LXFA 289, PRXF-MRI-H 1579, and LXFA 1041 tumors and 69% ΔTGI of PAXF 1657 tumors with greater activity seen at higher doses. Levels of MSLN expression assigned based on IHC in **A**.

To select appropriate *in vivo* tumor xenograft models to evaluate JNJ-79032421’s MSLN expression level–dependent cytotoxicity, first expression levels in primary normal and tumor MSLN-positive cells were determined. Membrane protein expression levels were determined by FACS analysis on single-cell suspensions. However, isolation of normal and cancer cells from tissues following enzymatic or mechanical dissociation methods led to loss of membrane MSLN. To remediate this, primary cells in suspension were collected from ascites and pleural effusions from patients with ovarian, pancreatic, and lung cancers. Matched FFPE solid tumor tissues from these patients were not available to assess MSLN expression by immunohistochemistry (IHC) staining, so correlation between MSLN expression by FACS and IHC could not be performed at this point. Serosal fluids contain a mixture of normal mesothelial cells derived from the pleural or intraperitoneal serosal lining, as well as cancer cells, immune cells, and other cell types (24). To distinguish the receptor densities on the normal mesothelial and cancer cells from the same patient in each sample, an 8-color FACS panel consisting of MSLN and other normal and cancer surface markers was designed. MSLN receptor densities in ovarian ascites and pleural effusions ranged from ∼500 to 6,200 receptors/cell on benign mesothelial cells and from ∼1,100 to 32,000 receptors/cell on cancer cells, confirming that tumor cells reach higher levels of MSLN than normal mesothelial cells (Fig. 3C). Based on these data, SK-OV-3 (7,800 receptors/cell), OVCAR-8 K1/C8 (45,000 receptors/cell), and OVCAR-8 (180,000 receptors/cell) cells, representing benign, medium, and high MSLN-expressing tumors, respectively, were selected for *in vivo* confirmation of JNJ-79032421 MSLN expression level–dependent cell killing activity. IHC on CDX tumors of these three models confirmed their low (benign), medium, and high MSLN expression *in vivo* (Fig. 4A).

### Influence of MSLN expression levels on JNJ-79032421 efficacy in human xenograft tumor models

Antitumor activities of JNJ-79032421 and MNC3B232 were assessed in the established human OVCAR-8 MSLN^HI+^, OVCAR-8 K1/C8 MSLN^MED+^, and SK-OV-3 MSLN^LO^ ovarian CDX models (Fig. 4B). Female NSG mice engrafted with human T cells bearing established subcutaneous CDX tumors were treated with JNJ-79032421. JNJ-79032421 (0.1 mg/kg) elicited significant antitumor efficacy over time against OVCAR-8 MSLN^HI+^ and OVCAR-8 K1/C8 MSLN^MED+^ CDXs with 173% and 150% ΔTGI, respectively, as compared with DPBS controls. At this dose, OVCAR-8 MSLN^HI+^ tumors regressed by 89% and 10 of 10 mice showed CR. For MSLN^MED+^ CDX tumors, 69% tumor regression was achieved and 2 of 10 showed CR. Treatment with JNJ-79032421 at dose levels <0.1 mg/kg did not result in significant antitumor activity in either xenograft model. In mice bearing SK-OV-3 MSLN^LO+^ tumors, treatment with JNJ-79032421 at 0.1 mg/kg resulted in a statistically, but not biologically significant response. In contrast, MNC3B232 treatment at 0.1 mg/kg did not elicit significant antitumor efficacy over time against OVCAR-8 MSLN^HI+^ or OVCAR-8 K1/C8 MSLN^MED+^ tumors, whereas it elicited significant antitumor efficacy leading to 99% ΔTGI against the SK-OV-3 MSLN^LO+^ model. JNJ-79032421 efficacy at 0.1 mg/kg was also observed in four PDX models with MSLN high-to-medium/low expression levels (Fig. 4C; Supplementary Table S4).

**Figure 5.**
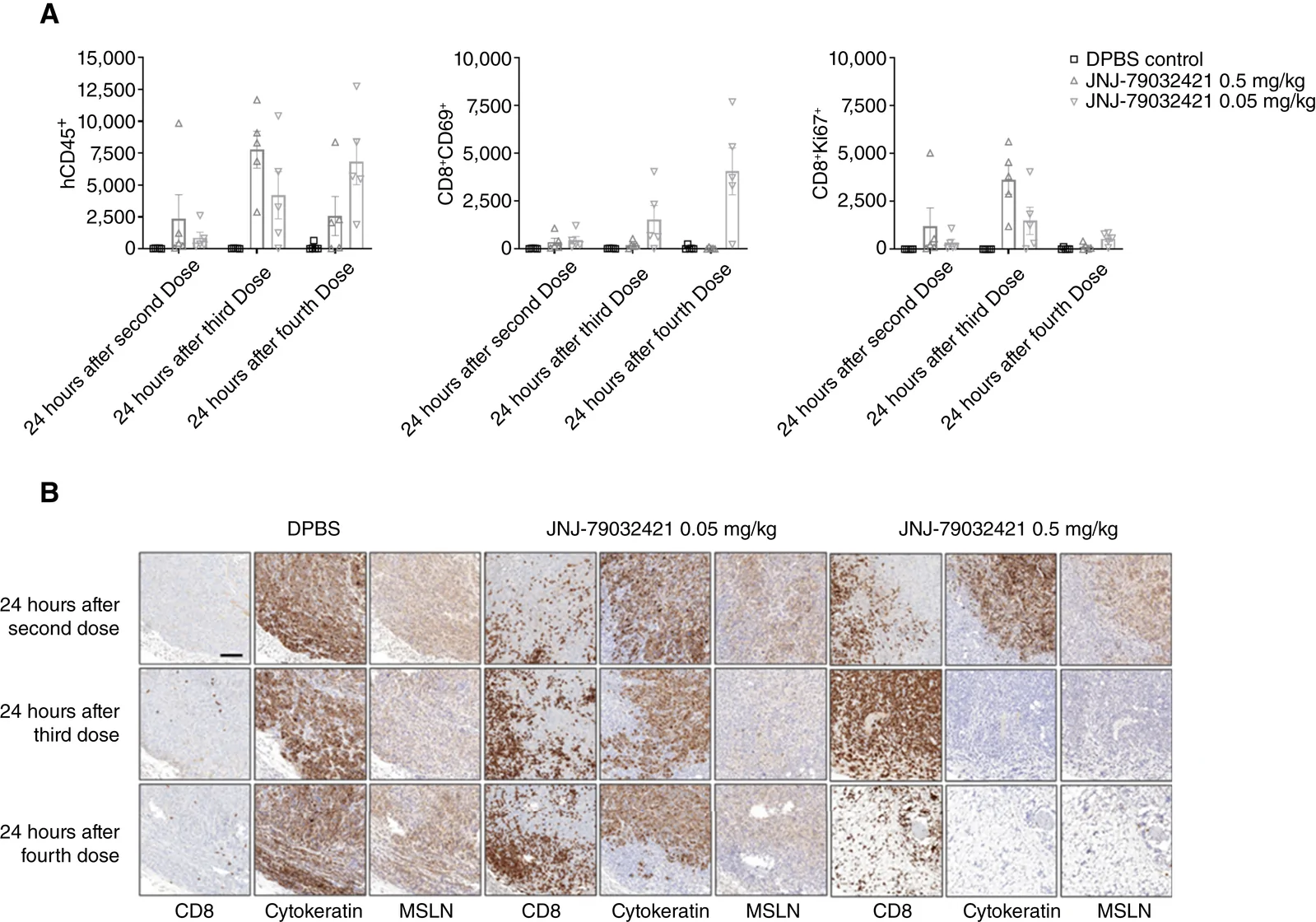
Dose- and time-dependent effects of JNJ-79032421 treatment on CD8^+^ and CD45^+^ T-cell infiltration and reduction of cytokeratin and MSLN expression. **A** and **B,** OVCAR-8 K1/C8 tumor cells (medium MSLN expression) were implanted on day 0, T cells were implanted on day 30, and treatment with JNJ-79032421 at 0.5 and 0.05 mg/kg occurred on days 31, 35, 38, and 42. Tumors were collected on days 36, 39, and 43 for **(A)** hCD45+ (counts normalized to tumor volume ±SEM) by flow cytometry analysis (*n* = 5 per group and per time point) and (**B**) IHC (*n* = 3 per group per time point) against CD8 (SP57), cytokeratin (AE1/AE3 + cytokeratin 8/18) and MSLN (5B2) performed on FFPE. Representative micrographs are shown for tumors treated as indicated and collected on day 36 (24 hours after second dose), day 39 (24 hours after third dose), and day 43 (24 hours after fourth dose). Sections were stained as indicated at the bottom of each column. Blue, cell nuclei (hematoxylin); brown, CD8, MSLN, or cytokeratin; magnification bar = 150 µm. hCD45, human cluster of differentiation 45.

The pharmacodynamic effect of JNJ-79032421 as determined by T-cell infiltration and activation was assessed in OVCAR-8 K1/C8 xenograft tumors by IHC and flow cytometry. At both 0.5 and 0.05 mg/kg, JNJ-79032421 inhibited tumor growth (Supplementary Fig. S3). Flow cytometry analysis of tumors treated with JNJ-79032421 at 0.5 mg/kg showed an increase in human CD45^+^ T cells after the second dose, with a peak following the third dose, and a decrease after the fourth dose of JNJ-79032421 (Fig. 5A). In tumors treated with JNJ-79032421 at 0.05 mg/kg, a gradual increase in human CD45^+^ T cells was observed over time. The CD8^+^ tumor-infiltrating lymphocytes (TIL) were proliferating, as shown by Ki67 positivity, peaking after the third dose and decreasing after the fourth dose, demonstrating the same trend as the TIL counts. Moreover, a gradual increase in CD69^+^ T-cell activation was observed in CD8^+^ TILs, particularly in animals treated with JNJ-79032421 at 0.05 mg/kg. In DPBS-treated tumors, minimal human CD45^+^ immune cell infiltration was detected.

In line with the flow cytometry data, IHC analysis demonstrated a dose-dependent increase in CD8^+^ T-cell infiltration in animals treated with JNJ-79032421 (Fig. 5B). At 0.5 mg/kg JNJ-79032421, CD8^+^ T-cell infiltration peaked following the third dose, whereas at 0.05 mg/kg the infiltration was higher following the fourth dose. Few T cells were observed in tumors treated with DPBS at these time points. As compared with OVCAR-8 K1/C8 tumors treated with DPBS, 0.5 mg/kg JNJ-79032421 treatment resulted in a time- and dose-dependent reduction of human MSLN- and cytokeratin-stained tumor cells and complete elimination of tumor cells after four doses, leaving a tissue mass consisting only of necrotic tumor debris, human T cells, and mouse inflammatory and stromal cells. These results demonstrated that treatment with JNJ-79032421 resulted in recruitment of T cells to the tumor, concurrent with tumor volume regression and histologic elimination of tumor cells.

## Discussion

Safe and effective targeting of solid tumors using antibody-mediated T-cell redirection therapy faces several challenges, including an immunosuppressive tumor microenvironment and normal tissue expression of target antigens. MSLN is a cancer lineage target that is expressed in mesothelial cells as well as in some epithelial cells of several tissues. Because MSLN can be highly expressed on cancer cells but suffers from a putative sMSLN “sink,” we developed antibodies against the membrane-restricted nonshed region of MSLN. As T cell–redirecting BsAbs are typically used at relatively low doses, the presence of such a soluble “sink” may potentially reduce their potency more than for modalities that use higher antibody concentrations, such as blocking antibodies or ADCs. Using an mrMSLN-targeting T-cell redirector is a novel approach to an extensively studied target that may enable efficacious antibody-mediated T-cell redirection to cancers (mesothelioma, ovarian cancer, and pancreatic cancer) with unmet medical need.

The hypothesis that sMSLN in the tumor microenvironment could impair the activity of MSLN-targeting antibody therapies by acting as a “sink” was supported by our observation of elevated blood levels of sMSLN, particularly in samples from patients with mesothelioma and ovarian cancers. In line with findings by Creaney and colleagues (25), serosal fluids of patients with cancer, in which normal mesothelial cells and circulating cancer cells can be found, contained high amounts of sMSLN (up to 90 nmol/L). Zhang and colleagues (13) demonstrated that sMSLN is likewise present in the tumor environment of pancreatic cancers, despite low sMSLN serum levels, suggesting that circulating sMSLN levels may underestimate the level of sMSLN in the tumor microenvironment. In addition, our data further established that tumors from various cancer types, including mesothelioma and serous ovarian, pancreatic, and lung cancers, have high concentrations of sMSLN in the tumor microenvironment, often surpassing the levels of full-length MSLN presented on the membrane.

JNJ-79032421 was developed to target the membrane-restricted region of MSLN to avoid the sMSLN “sink” generated by enzymes shown to cleave MSLN between residues 583 and 592. Indeed, JNJ-79032421 was shown to bind to full-length MSLN (K_D_ = 4.64 nmol/L) but not to recombinant sMSLN lacking the seven most C-terminal amino acids (SMQEALS). Binding of JNJ-79032421 to the GPI-anchored 12aa MSLN stub remaining on the membrane after sMSLN shedding (residues 587–598; LVLDLSMQEALS) was very weak, as evidenced from the binding EC_50_ > 100 nmol/L and only threefold reduction of the binding EC_50_ to OVCAR-8 cells in the presence of 1 µmol/L 12aa peptide, suggesting that JNJ-79032421–induced T cell–mediated killing acts through targeting of the full-length MSLN only. Both structural and functional data demonstrated MSLN residues Leu589, Asp590, and Met593 (not present on sMSLN) as critical residues required for JNJ-79032421 MSLN binding. Based on the co-crystal structure, JNJ-79032421 binds to a helical region that may not be maintained in the 12aa peptide and the GPI-12aa peptide expressing OVCAR-8 cells. An interaction involving Gly585, an amino acid outside the 12aa peptide, could also be required for full binding.

Targeting mrMSLN translated into evidence of biological activity. JNJ-79032421 displayed expression level–dependent T cell–mediated cytotoxicity in a broad panel of cancer cell lines (*n* = 17) with MSLN expression levels ranging from 1,700 to 490,000 receptors/cell. In contrast, the activity of nmrMSLNxCD3 MNC3B232, binding to both membrane MSLN and sMSLN, was not dependent on MSLN cell expression density. We hypothesize that sMSLN released from cells in the medium reduced MNC3B232’s T cell–mediated cytotoxicity to a higher extent in high MSLN-expressing than in low MSLN-expressing cells. It should be noted that the BsAbs used in these studies differed in affinity and that the higher affinity of MNC3B232 likely contributed to the reduction of potency in high MSLN-expressing cells. Killing assays showed that lower-affinity nmrMSLNxCD3 MNC3B597 was less susceptible to competition with sMSLN and showed some correlation with receptor density, albeit to a lesser extent than JNJ-79032421 with similar affinity. This observation is consistent with published data showing that an affinity-tuned CAR-T utilizing a low-affinity nmrMSLN binder (K_D_ >100 nmol/L) discriminated between low, medium, and high expression (26). Although tuning the affinity of nmrMSLN binders could be a strategy to reduce the effect of sMSLN, our data demonstrated that targeting the membrane-restricted region of MSLN completely eliminates the inhibitory effects of sMSLN, especially in the tumor microenvironment in which concentrations are the highest, a hypothesis that was further substantiated by *in vivo* CDX efficacy studies.

*In vivo* efficacy data in MSLN^LO+/MED+/HI+^ CDX models confirmed that JNJ-79032421’s potency increased with increasing MSLN expression levels. Indeed JNJ-79032421 treatment led to significant antitumor efficacy in MSLN^MED+^ and MSLN^HI+^ tumor models, whereas its activity was lower in the MSLN^LO+^ model, a surrogate for normal MSLN tissue expression. Although this may reduce toxicity against normal tissues with low MSLN expression, it is also possible that tumors with heterogeneous and low MSLN expression may be able to escape JNJ-79032421 treatment. For this reason, patient selection criteria, including MSLN expression levels, may be helpful to explore this question. Conversely, nmrMSLNxCD3 MNC3B232 demonstrated decreasing activity with increasing MSLN expression to a greater extent *in vivo* than in *in vitro* tumor models. A possible explanation is that sMSLN increases from the MSLN ^LO+^ to MSLN ^MED+^ to MSLN ^HI+^ cells and diffuses more slowly in the tumor microenvironment than in culture media. *In vivo*, sMSLN concentrations in proximity of the cell membrane may remain at higher concentration depending on tumor vascularization. As a result, anti-nmrMSLN BsAbs may be more active against normal tissues with low MSLN expression (low sMSLN “sink”), especially if their affinity for MSLN is high, and may lose efficacy in cancer tissues with high MSLN expression (high sMSLN “sink”).

We also demonstrated that JNJ-79032421 antitumor treatment resulted in recruitment of T cells to the tumor, concurrent with elimination of tumor cells. JNJ-79032421 antitumor activity was furthermore demonstrated in PDX models of pancreatic, lung, and ovarian cancers.

Taken together, our preclinical data show that JNJ-79032421 binds in a unique and unprecedented manner to MSLN. JNJ-79032421 binds mrMSLN (and not sMSLN) and is highly efficacious (*in vitro* and *in vivo*) against medium and high MSLN-expressing cancer cells but spares low MSLN-expressing cancer cells, which we believe are a reasonable surrogate for normal MSLN-expressing tissues. Unlike MSLNxCD3 BsAbs capable of binding both full-length membrane-bound and sMSLN, JNJ-79032421 *in vitro* and *in vivo* activity is unaffected by the presence of sMSLN, which we demonstrated to be present at high levels in the tumor microenvironment. The differentiated targeting of JNJ-79032421 may increase its therapeutic potential, particularly against high/medium MSLN-expressing tumors, while sparing normal/low-expressing tissues. In summary, JNJ-79032421 demonstrates a favorable preclinical efficacy profile, supporting its current safety and tolerability investigation in a phase I clinical study (NCT06255665).

## Supplementary Material

Figure S1 Figure S1 shows the CD3 W245LH EC50 binding to T-cells as determined by flow cytometry in 3 individual donors.

Figure S2 Figure S2 shows the 2.0-Å crystal X-ray structure and epitope determination of MSLNB703 Fab bound to the C-terminal peptide of human MSLN

Figure S3 Figure S3 shows individual tumor volumes upon 4 treatments with DPBS or 0.5 or 0.05 mg/kg JNJ-79032421 in the mouse OVCAR-8 K1/C8 CDX model.

Table S1 Table S1 provides the heavy and light variable chain sequence information for MSLN and CD3 antibodies used

Table S2 Table S2 contains a list of all cell lines used with associated information

Table S3 Table S3 summarizes the fold change of geomean killing EC50 values (with sMSLN versus without sMSLN) for mrMSLNxCD3 and nmrMSLNxCD3 BsAbs

Table S4 Table S4 the efficacy (tumor growth inhibition values) of JNJ-79032421 with MSLN expression levels in PDX mouse models

## Data Availability

Data were generated by the authors and is available on request. The X-ray structure was deposited in the PDB database (PDB ID 9Z3B).
