# Supplementary material for: JNJ-79032421, a Novel Membrane-Restricted Mesothelin-Targeting T Cell–Engaging Bispecific Antibody for the Treatment of Mesothelin-Positive Cancers
Source: Mol Cancer Ther. 2026 Mar 3;25(10):1585–98. doi: 10.1158/1535-7163.MCT-25-0665 (PMC13631496; doi:10.1158/1535-7163.MCT-25-0665)
Supplement: Supplementary file 1 [file mct-25-0665_figure_s1_suppsf1.pdf]

Figure S1: CD3 W245LH EC50 as determined by flow cytometry using T-cells from 3 donors. The isotype control did not show any binding.

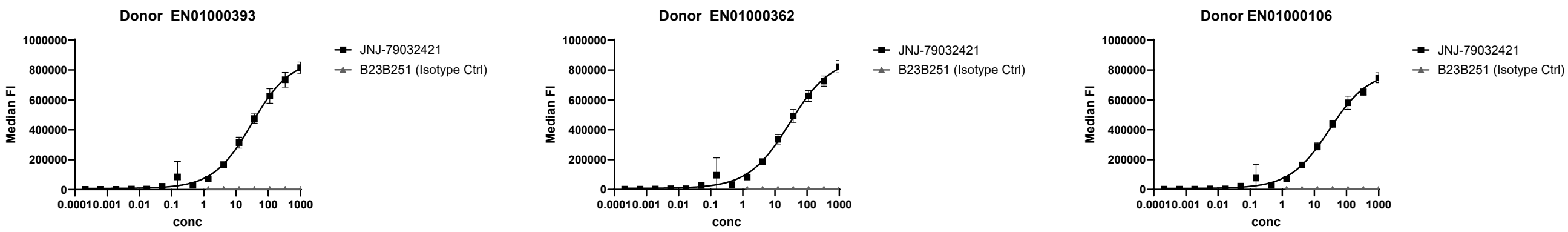

EC50=30.41

EC50=28.60

EC50=29.21
