# Supplementary material for: JNJ-79032421, a Novel Membrane-Restricted Mesothelin-Targeting T Cell–Engaging Bispecific Antibody for the Treatment of Mesothelin-Positive Cancers
Source: Mol Cancer Ther. 2026 Mar 3;25(10):1585–98. doi: 10.1158/1535-7163.MCT-25-0665 (PMC13631496; doi:10.1158/1535-7163.MCT-25-0665)
Supplement: Supplementary file 2 [file mct-25-0665_figure_s2_suppsf2.pdf]

Figure S2: X-ray structure and epitope determination.

A B

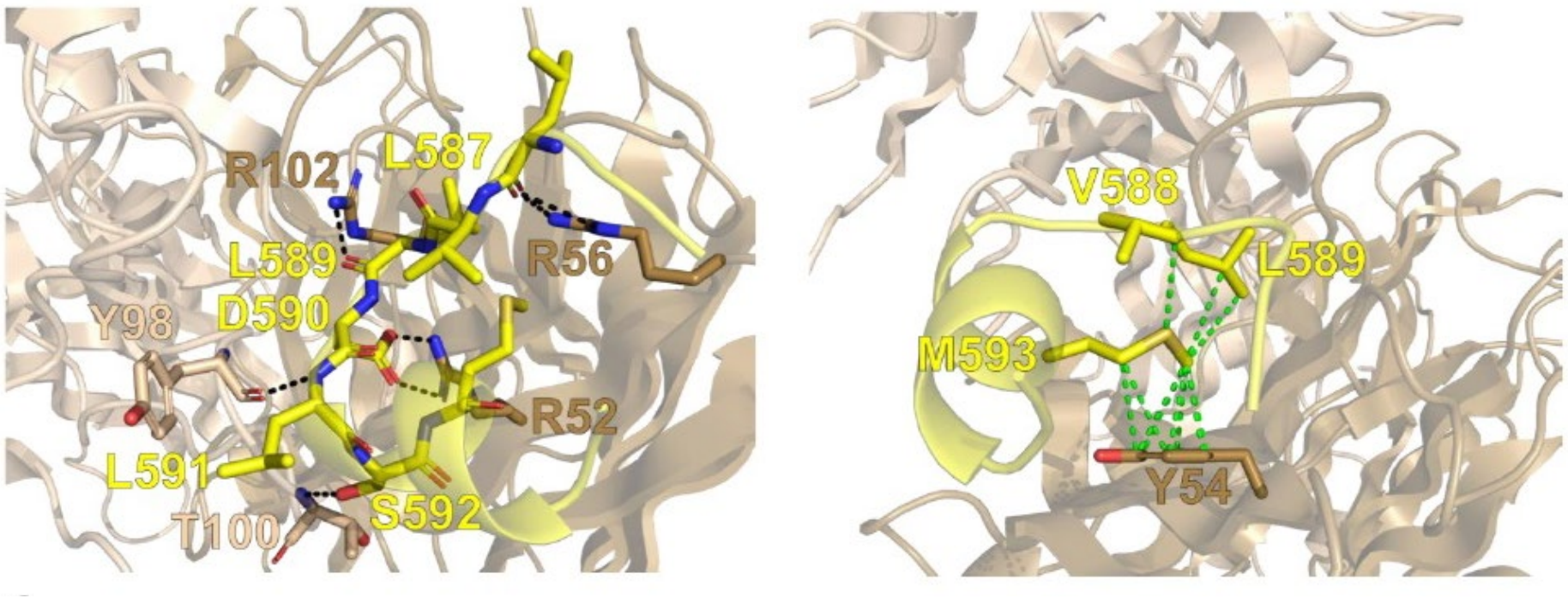

Fab, fragment antigen-binding; HV, heavy variable; MSLN, mesothelin.

The 2.0-Å crystal structure of MSLNB703 Fab bound to the C-terminal peptide of human MSLN. The MSLN peptide is represented as sticks colored in yellow. (A) Residues involved in ionic and hydrogen bonding (black dashed lines) between MSLNB703 and MSLN are shown as sticks and labeled. (B) The side chain of MSLN residue Met593 is buried into a hydrophobic sub-pocket formed upon Fab-peptide complex formation lined with Val588 and Leu589 of MSLN and against the side chain of MSLNB703 HV residue Tyr54.
