# Supplementary material for: JNJ-79032421, a Novel Membrane-Restricted Mesothelin-Targeting T Cell–Engaging Bispecific Antibody for the Treatment of Mesothelin-Positive Cancers
Source: Mol Cancer Ther. 2026 Mar 3;25(10):1585–98. doi: 10.1158/1535-7163.MCT-25-0665 (PMC13631496; doi:10.1158/1535-7163.MCT-25-0665)
Supplement: Supplementary file 3 [file mct-25-0665_figure_s3_suppsf3.pdf]

Figure S3: Individual tumor volumes upon 4 treatments with DPBS or 0.5 or 0.05 mg/kg JNJ-79032421 in OVCAR-8 K1/C8 CDX tumors.

A.

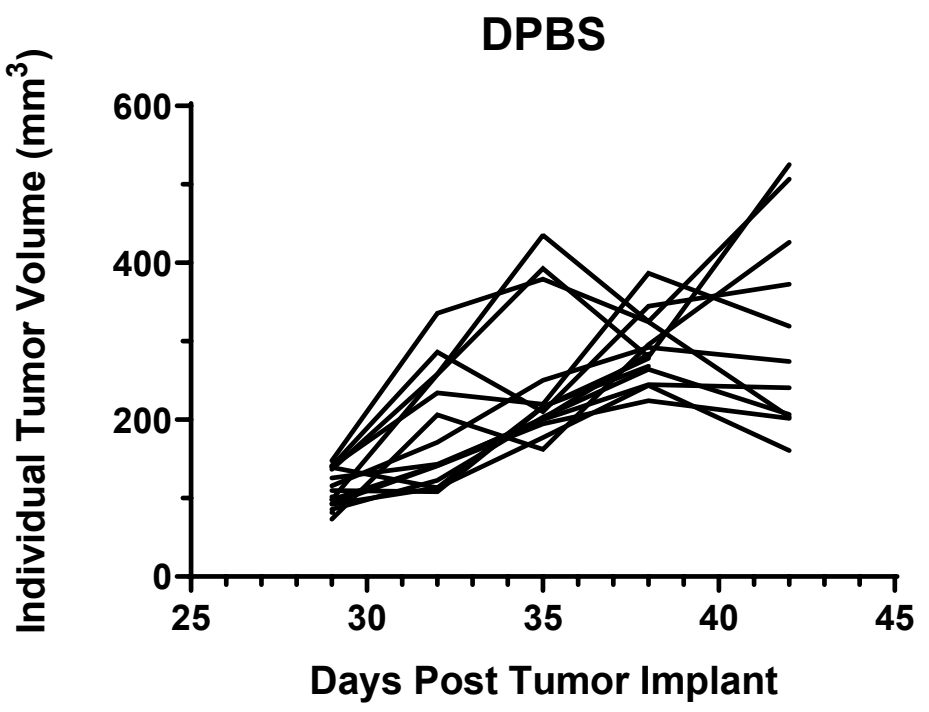

B.

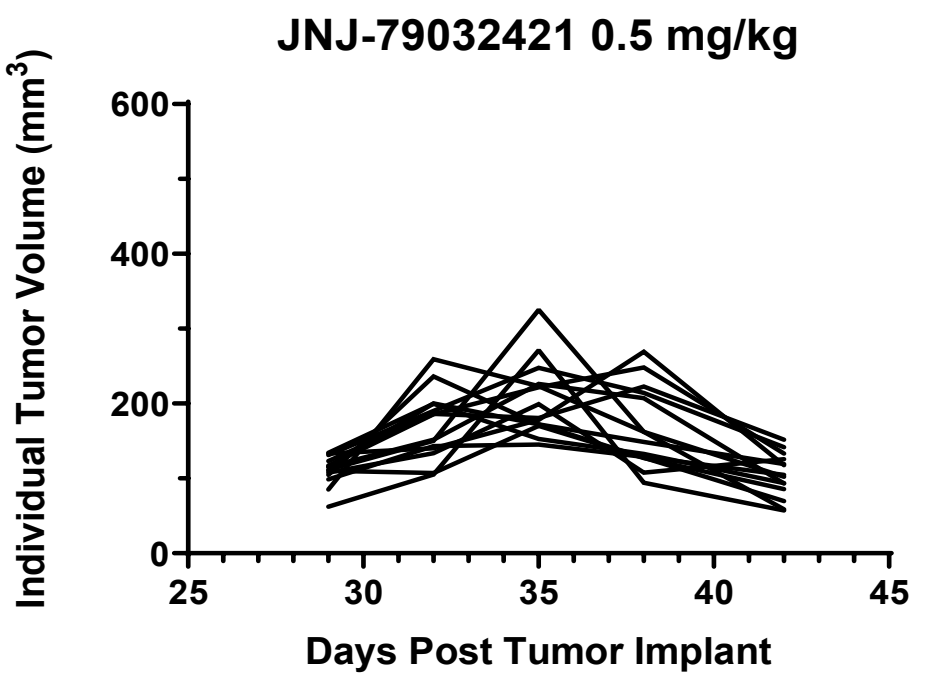

C.

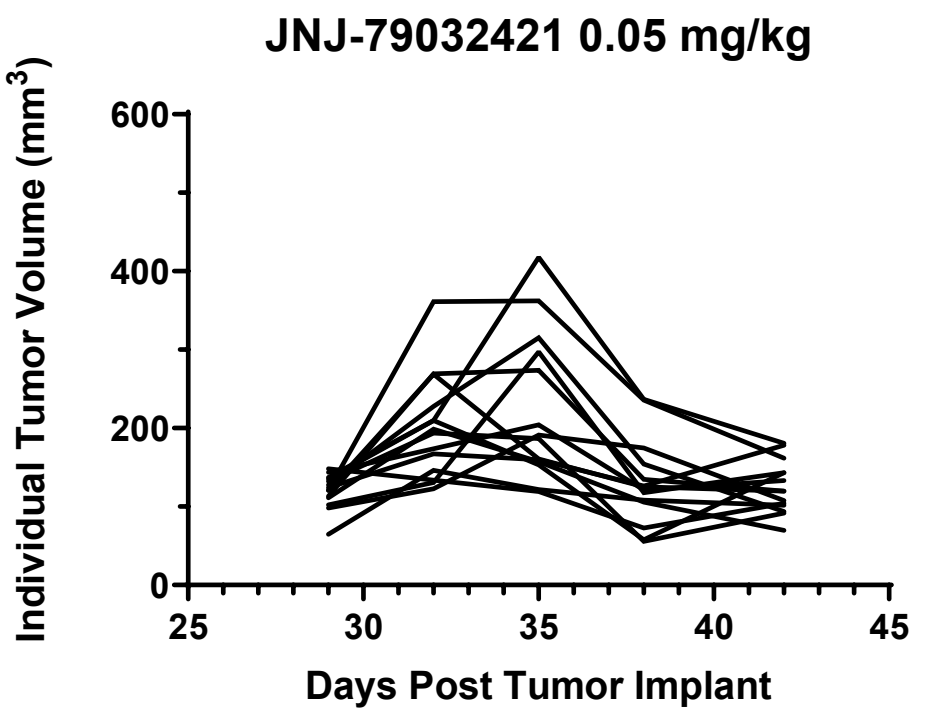

CDX, cell-line-derived xenograft; PBS, phosphate-buffered saline.

Individual tumor volumes (n=11/group) over time in OVCAR-8 K1/C8 CDX model (A) PBS, (B) 0.5 mg/kg JNJ-79032421, (C) 0.05 mg/kg JNJ-79032421. Tumor cells were implanted on Day 0. Mice were humanized with T cells on the day of randomization and treatment was initiated 1 day after T cell implantation, Days 31, 35, 38, and 42.
