# Supplementary material for: JNJ-79032421, a Novel Membrane-Restricted Mesothelin-Targeting T Cell–Engaging Bispecific Antibody for the Treatment of Mesothelin-Positive Cancers
Source: Mol Cancer Ther. 2026 Mar 3;25(10):1585–98. doi: 10.1158/1535-7163.MCT-25-0665 (PMC13631496; doi:10.1158/1535-7163.MCT-25-0665)
Supplement: Supplementary file 4 [file mct-25-0665_table_s1_suppst1.pdf]

Table S1: Sequences non-commercial MSLN and CD3 antibodies

Name	Isotype	HC VR Sequence	LC VR Sequence
MSNB457	IgG1, Kappa	QVQLVESGPGQVKPSQTLSLTCAISGDSVSSNSAAWNWIRQSPSRGLEWLGRTYYRSKWYNDYAVSVKSRITINPDTSKNQFSLQLNSVTPEDTAVYYCARRDYSSGGFDYWGQGTTVTVSS	EIVLTQSPDSLAVSLGERATINCKSSQSVLYSSDNKNYLAWYQQKPGQPPNLLIYWASTRESGVPDRFSGSGSGTDFTLTINSLQAEDVAVYYCQQYYSTPFTFGPGTKVEIK
MSLNB570	IgG1, Kappa	QVQLQQSGPGLVKPSQTLSLTCAISGDSVSSTSVAWNWIRQSPSGGLEWLGRTYYRSKWYNDYALSVKSRMTINPDTASNQFSLQLNSVTPEDTAVYYCSRGIVGALDYWGQGTLVTVSS	DIVMTQTPLSLPVTPGGPASISCRSSQSLMYSNGYNHLDWYLQKPGQSPQFLIHLGSNRVSGVPDRFSGSGSGTDFTLKISRVEAEDVGVYYCMQALRTPYTFGQGTKVDIK
MSNB71	IgG1, Kappa	QVQLQQSGPGLVMSSQTLSLTCAISGDSVSSSSVAWNWIRQSPSRGLQWLGRTYFRSKWYYDYAVSVKSRITINPDTSKNQFSLQLNSVTPEDTAVYYCSRGIVGALDYWGQGTLVTVSS	DIVMTQSPLSLPVTPGEPASISCRSSQSLLFTNGYDSLDWYLQKPGQSPQLLIYLVSNRASGVPDRFSGSGSGTEFTLKISRVEADDVGVYFCMQGIRTPFTFGPGTKLEIK
MSNB95	IgG1, Kappa	QVQLQESGPGLVKPSETLSLTCTVSGGSVSSYYWSWIRQPPGKGLEWIGYFFYSGSSNYNPSLKSRVTISLDTSKNQVSLKLSSVTAADTAVYYCARGGPTGFAFDIWGQGTMVTVSS	DIVMTQSPSAMSASVGDRVTITCRASQGISNYLAWFQQKPGKVPKRLIYAASSLQSGVPSRFSGSGSGTEFTLTISRLQPEDFATYYCLQHNSFPFTFGQGTKVDIK
MSNB90	IgG1, Kappa	QVQLQESGPGLVKPSETLSLTCTVSGGSIRSYHWNWIRQSAGKGLEWIGRFSTSGTTNSNPSLKSRVTMSVDTSKNQFSLKLTSVTAADTAVYYCAGTDAFDIWGQGTMVTVSS	DIQMTQSPSAMSASVGDRVTITCRASQVISNYLAWFQQKPGKVPKRLIYAASGLQSGVPSRFSGSGSGTEFTLTVSSLQPEDFATYYCLQHYSFPRTFGQGTKVDIK
MSNB9	IgG1, Kappa	QVQLQESGPGLVKPSQTLSLTCTVSGGSINNNNYYWTWIRQHPGKGLEWIGYIYYSGSTFYNPSLKSRVTISVDTSKTQFSLKLSSVTAADTAVYYCAREDTMTGLDVWGQGTTVTVSS	DIQMTQSPSSLSASVGDRVTITCRASQSINNYLNWYQQKPGKAPTLLIYAASSLQSGVPSRFSGSRSGTDFTLTISSLQPEDFAAYFCQQTYSNPTFGQGTKVEVK
CD3W245	IgG1, Kappa	EVQLVESGGGLVKPGGSLRLSCAASGFTFSRYNMNWVRQAPGKGLEWVSSISTSSNYIYYADSVKGRFTFSRDNAKNSLDLQMSGLRAEDTAIYYCTRGWGPFDYWGQGTLVTVSS	DIQMTQSPSSLSASVGDRVTITCRARQSIGTAIHWYQQKPGKAPKLLIKYASESISGVPSRFSGSGSGTDFTLTISSLQPEDFATYYCQQSGSWPYTFGQGTKLEIK

HC heavy chain; LC light chain; VR variable region
