# Supplementary material for: JNJ-79032421, a Novel Membrane-Restricted Mesothelin-Targeting T Cell–Engaging Bispecific Antibody for the Treatment of Mesothelin-Positive Cancers
Source: Mol Cancer Ther. 2026 Mar 3;25(10):1585–98. doi: 10.1158/1535-7163.MCT-25-0665 (PMC13631496; doi:10.1158/1535-7163.MCT-25-0665)
Supplement: Supplementary file 5 [file mct-25-0665_table_s2_suppst2.pdf]

Table S2: Cancer cell lines used in experiments.

Cell line	Tissue source (human)	Source	ABC (MSN B457-PE)	Complete culture medium	Sex	Acquisition date	Myco Plasma/ Authentication	Passage number used
Capan-2	Pancreatic adenocar-cinoma	ATCC HTB-80	29,000	McCoy's 5A, 10% FBS, 2 mM L-glutamine, 50 µg/mL gentamycin	M	3/4/ 2019	3/4/2019 6/17/21	6
Capan-2 NucLight Red	Pancreatic adenocar-cinoma	Engineered cell line from Capan-2	See Capan-2	McCoy's 5A, 10% FBS, 2 mM L-glutamine, 50 µg/mL gentamycin, 1 µg/mL puromycin	M	N/A$	NA 6/17/21	2 and 5
COR-L23	Large cell lung carcinoma	ECACC 92031919	77,000	RPMI 1640, 10% FBS, 2 mM glutamine, 50 µg/mL gentamycin	M	9/05/ 1997	10/16/2016 NA	2 and 4
MDA-MB-231	Breast adenocar-cinoma	ATCC HTB-26	9,600	DMEM, 10% FBS, 2 mM L-glutamine, 1 mM sodium pyruvate, 50 µg/mL gentamycin	F	10/25/ 1995	6/9/2023 6/9/2023	3 and 4
MeT-5A	Lung mesotheli oma	ATCC HTB-26	15,000	Medium 199, 10% FBS, 2 mM L-glutamine, 10 mM HEPES, 3.3 nM EGF, 400 nM hydrocortisone, 870 nM insulin, 1% pen/strep, 0,1% amphotericin B	M	2/15/ 2018	2/15/2018* 2/15/2018*	2 and 5
MSTO-211H	Lung mesotheli oma	ATCC CRL-2081	1,700	RPMI 1640, 10% FBS, 2 mM glutamine, 50 µg/mL gentamycin	M	10/15/ 2020	10/15/2020* 10/15/2020*	8
NCI-H292	Mucoepi dermoid lung carcinoma	ATCC CRL-1848	17,000	RPMI 1640, 10% FBS, 2 mM glutamine, 10 mM HEPES, 1 mM sodium pyruvate, 4.5 g/L glucose, 50 µg/mL gentamycin	F	11/16/ 2006	9/7/2005* 6/17/2021	10
NCI-H292 NucLight Red	Mucoepi dermoid lung carcinoma	Engineered cell line from NCI-H292	See NCI-H292	RPMI 1640, 2 mM glutamine, 10% FBS, 10 mM HEPES, 1 mM sodium pyruvate, 4.5 g/L glucose, 50 µg/mL gentamycin, 1 µg/mL puromycin	F	N/A$	NA 6/17/2021	2 and 5
NCI-H838	NSCLC	ATCC CRL-5844	150,000	RPMI 1640, 10% FBS, 2 mM glutamine, 10 mM HEPES, 1 mM sodium pyruvate, 4.5 g/L glucose, 50 µg/mL gentamycin	M	03/30/ 2005	7/26/2019 03/30/2005*	3 and 7

Cell line	Tissue source (human)	Source	ABC (MSN B457-PE)	Complete culture medium	Sex	Acquisition date	Myco Plasma/ Authentication	Passage number used
SK-OV-3	Ovarian adenocar-cinoma	ATCC HTB-77	7,800	McCoy's 5A, 10% FBS, 2 mM L-glutamine, 50 µg/mL gentamycin	F	6/21/ 2000	10/9/2019 10/402019	2 and 3
OVCAR-8	Ovarian adenocar-cinoma	National Cancer Institute	180,000	RPMI 1640, 10% FBS, 2 mM glutamine, 50 µg/mL gentamycin	F	8/6/ 2019	9/12/2019 9/10/2019	5, 8 and 13
OVCAR-8 NucLight Red	Ovarian adenocar-cinoma	Engineered cell line from OVCAR-8	See OVCAR-8	RPMI 1640, 10% FBS, 2 mM glutamine, 50 µg/mL gentamycin, 1 µg/mL puromycin	F	N/A[$]	9/12/2019 9/10/2019	2 and 5
OVCAR-8 LM2/B6	Ovarian adenocar-cinoma	Engineered cell line from OVCAR-8	490,000	RPMI 1640, 10% FBS, 2 mM glutamine, 1 mM sodium pyruvate, 1% NEAA, 50 µg/mL gentamycin, 0.5 µg/mL puromycin, 0.2 mg/mL geneticin	F	5/10/ 2019	5/10/2019* 7/23/19	7
OVCAR-8 K1/C8	Ovarian adenocar-cinoma	Engineered cell line from OVCAR-8	45,000	RPMI 1640, 10% FBS, 2 mM glutamine, 1 mM sodium pyruvate, 1% NEAA, 0.2 mg/mL geneticin	F	5/24/ 2019	1/25/2021 1/18/2021	7, 9 and 11
OVCAR-8 GPI-12aa C-term	Ovarian adenocar-cinoma	Engineered cell line from OVCAR-8	N/A	RPMI 1640, 10% FBS, 2 mM glutamine, 1 mM sodium pyruvate, 1% NEAA, 0.2 mg/mL geneticin	F	2/28/ 2020	2/28/2020* 7/23/19	7
HNXF 1859L[a]	Head and neck cancer	CRL	5,600	RPMI 1640, 10% FBS, 2 mM glutamine, 10 mM HEPES, 1 mM sodium pyruvate, 1% NEAA, 1% pen/strep	F	8/10/ 2020	8/10/2020* N/A	4 and 5
LXFA 983L[a]	Lung adenocar-cinoma	CRL	7,500	RPMI 1640, 10% FBS, 2 mM glutamine, 10 mM HEPES, 1 mM sodium pyruvate, 1% NEAA, 1% pen/strep	M	8/10/ 2020	8/10/2020* N/A	2, 3 and 9
OVXF 1023L[a]	Ovarian adenocar-cinoma	CRL	1,900	RPMI 1640, 10% FBS, 2 mM glutamine, 10 mM HEPES, 1 mM sodium pyruvate, 1% NEAA, 1% pen/strep	F	8/10/ 2020	8/10/2020* N/A	2 and 9
LXFA 289	NSCLC	CRL	N/A	Tumor fragments	M	N/A**	9/16/2021 N/A	
PAXF 1657	Pancreatic cancer	CRL	N/A	Tumor fragments	F	N/A**	1/27/2022 N/A	
PRXF-MRI-H 1579	Prostate cancer	CRL	N/A	Tumor fragments	M	N/A**	9/16/2021 N/A	

Cell line	Tissue source (human)	Source	ABC (MSN B457-PE)	Complete culture medium	Sex	Acquisition date	Myco Plasma/ Authentication	Passage number used
LXFA 1041	NSCLC	CRL	N/A	Tumor fragments	M	N/A**	9/16/2021 N/A	

12aa, 12 amino acid; ABC, antibody binding capacity; ATCC, American Type Culture Collection; CRL, Charles River Laboratories; C-term, C-terminal; DMEM, Dulbecco's modified Eagle medium; ECACC, European Collection of Authenticated Cell Cultures; EGF, epidermal growth factor; FBS, fetal bovine serum; HEPES, 4-(2-hydroxyethyl)-1-piperazineethanesulfonic acid; GPI, glycosylphosphatidylinositol; MSLN, mesothelin; N/A, not applicable; NA, not available; NEAA, non-essential amino acids; NSCLC, non-small-cell lung carcinoma; PE, phycoerythrin; pen/strep, penicillin/streptomycin; RPMI, Roswell Park Memorial Institute;

ABC values for each cell line are rounded. [$]NucLightRed cell lines were generated in house. They express red fluorescent protein mKate2, which interfered with ABC determination; MSLN ABC values for these cell lines were therefore adopted from the respective parental cell lines. The OVCAR-8 K1/C8 and LM2/B6 cell lines were derived from the parental OVCAR-8 cell line by deletion of MSLN followed by reintroduction of MSLN and clonal selection of the desired level of MSLN expression. The OVCAR-8 GPI-12aa C-term cell line was derived from the parental OVCAR-8 cell line by deletion of MSLN followed by reintroduction of the MSLN sequence coding the 12aa MSLN C-terminus. [a] Denotes patient-derived xenograft cells. *Denotes date is from freezing stock provider, and not retested in house

N/A** Acquisition date not applicable as PDX fragments (models derived from patient tissues generated at CRL, Freiburg) were used in in vivo PDX efficacy studies at CRL and were not acquired by Johnson & Johnson to be used in house.
