# Supplementary material for: JNJ-79032421, a Novel Membrane-Restricted Mesothelin-Targeting T Cell–Engaging Bispecific Antibody for the Treatment of Mesothelin-Positive Cancers
Source: Mol Cancer Ther. 2026 Mar 3;25(10):1585–98. doi: 10.1158/1535-7163.MCT-25-0665 (PMC13631496; doi:10.1158/1535-7163.MCT-25-0665)
Supplement: Supplementary file 6 [file mct-25-0665_table_s3_suppst3.pdf]

Table S3: Fold change of geomean killing EC50 values (nM) for mrMSLNxCD3 and nmrMSLNxCD3 BsAbs.

	**sMSLN (nM)**											
	**0**	**1**	**10**	**100**	**0**	**1**	**10**	**100**	**0**	**1**	**10**	**100**
	**OVCAR-8 (72h)**				**OVCAR-8 K1/C8 (72h)**				**SK-OV-3 (48h)**			
MNC3B321 (B9)	1	3.447	23.349	283.323	1	15.052	132.465	1401.315	1	23.01	70.36	
MNC3B319 (B71)	1	0.934	1.451	0.974	1	0.666	0.571	0.468	1	0.77	0.88	1.10
MNC3B597 (B95)	1	1.015	2.587	14.693	1	0.880	2.796	14.682	1	0.62	0.74	
JNJ-79032421 (B457)	1	1.032	1.224	1.083	1	0.946	1.003	0.815				

BsAb, bispecific antibody; EC50, 50% effective concentration; sMSLN, shed mesothelin.
