# Supplementary material for: JNJ-79032421, a Novel Membrane-Restricted Mesothelin-Targeting T Cell–Engaging Bispecific Antibody for the Treatment of Mesothelin-Positive Cancers
Source: Mol Cancer Ther. 2026 Mar 3;25(10):1585–98. doi: 10.1158/1535-7163.MCT-25-0665 (PMC13631496; doi:10.1158/1535-7163.MCT-25-0665)
Supplement: Supplementary file 7 [file mct-25-0665_table_s4_suppst4.pdf]

Table S4: Correlation of efficacy of JNJ-79032421 with MSLN expression levels in PDX models.

PDX model	Organ	MSLN	ΔTGI at 2.5, 0.5, 0.1, 0.05 mg/kg
LXFA 289	Lung	High	97%, 98%, 106%, 93% (Day 39)
PAXF 1657	Pancreas	Medium/high	107%, 101%, 69%, 26% (Day 31)
PRXF-MRIH 1579	Prostate	Medium	112%, 112%, 110%, 86% (Day 39)
LXFA 1041	Lung	Medium/low	126%, 126%, 102%, 67% (Day 38)

MSLN, mesothelin; PDX, patient-derived xenograft; SEM, standard error of the mean; TGI, tumor growth inhibition.

ΔTGI values upon treatment of JNJ-79032421 in PDX mouse models. Tumor cells were implanted on Day 0. Mice were humanized with T cells on the day of randomization and treatment was initiated 1 day after T cell implantation. Mice were treated IP twice weekly for 3 weeks on Days 19, 22, 26, 29, 33, and 36 (LXFA 289 $MSLN^{HI+}$ and PRXF-MRIH 1579 $MSLN^{MED+}$), Days 18, 21, 25, 28, 32, and 35 (PAXF 1657 $MSLN^{MED+/HI+}$ and LXFA 1041 $MSLN^{MED+/LO+}$).
